# Translational Profiling of Drd2-Expressing Populations Reveals Molecular Heterogeneity of Dentate Gyrus Mossy Cells along the Dorsoventral Axis

**DOI:** 10.1523/ENEURO.0236-25.2026

**Published:** 2026-07-14

**Authors:** Minseok Jeong, Jin-Hyeok Jang, Seo-Jin Oh, Ji-Woong Choi, Yong-Seok Oh

**Affiliations:** ^1^Department of Brain Sciences, Daegu-Gyeongbuk Institute of Science and Technology, Daegu 42988, Republic of Korea; ^2^Department of Electrical Engineering and Computer Science, DGIST, Daegu 42988, Republic of Korea; ^3^Brain Engineering Convergence Research Center, DGIST, Daegu 42988, Republic of Korea; ^4^Emotion, Cognition & Behavior Research Group, Korea Brain Research Institute, Daegu 41062, Republic of Korea

**Keywords:** dentate gyrus, dorsoventral axis, heterogeneity, hippocampus, mossy cells, translating ribosome affinity purification, translatome

## Abstract

Hilar mossy cells (MCs) are crucial for integrating and propagating signals across the hippocampal dorsoventral axis, mediating cognitive and affective processing. While MCs exhibit profound dorsoventral differences in their projections, physiology, and behavioral roles, the molecular basis underlying this functional specialization remains largely unexplored. To address this gap, we used translating ribosome affinity purification (TRAP) in male mice to systematically compare the translatome of *Drd2*-expressing, MC-enriched populations along the dorsoventral axis. This analysis revealed distinct translational signatures with 1,442 genes enriched in dorsal and 1,337 genes in ventral *Drd2*-expressing, MC-enriched populations. Pathway analysis demonstrated significant functional segregation along the dorsoventral axis. The dorsal population is notably enriched for genes linked to neuronal connectivity and synaptic transmission, whereas the ventral counterpart shows enrichment in genes associated with energy metabolism and cellular maintenance. Specifically, we identified a subset of dorsal enriched genes, including neurotransmitter receptors, ion channels, and axon guidance regulators, contrasting with ventral enriched genes highly related to glucose/fatty acid metabolism, oxidative phosphorylation, and exocytosis. We further predicted distinct sets of upstream transcriptional regulators activated in each subpopulation, providing insights into the regulatory networks that may drive molecular divergence. Our findings provide a translatomic basis for the dorsoventral heterogeneity of Drd2-expressing neurons that include MCs, offering molecular signatures associated with their differential contributions to hippocampal function.

## Significance Statement

Mossy cells (MCs) are crucial for integrating signals and propagating them across the hippocampal dorsoventral axis, influencing both cognitive and emotional processing. Despite functional differences, the molecular basis of dorsoventral heterogeneity has remained elusive. Utilizing TRAP analysis with *Drd2*-Cre-driven EGFP-L10a labeling, our study uncovers distinct translational signatures within *Drd2*-expressing, MC-enriched populations along the dorsoventral axis. Specifically, the dorsal population is genetically poised for enhanced neuronal signaling and intrinsic excitability, whereas the ventral population preferentially expresses genes that support robust energy metabolism and cellular resilience. These findings advance our understanding of how distinct molecular features may underlie dorsoventral functional specialization of MCs, providing insights into circuit mechanisms supporting cognitive and affective behaviors and their potential differential vulnerability in brain disorders.

## Introduction

The hippocampus, a limbic structure essential for learning, memory, and emotional regulation, is functionally specialized along its dorsoventral (longitudinal) axis (Moser and Moser, 1998; Fanselow and Dong, 2010; Strange et al., 2014). While the dorsal hippocampus is primarily engaged in spatial navigation and cognitive processing, the ventral hippocampus predominantly supports anxiety, stress responses, and affective behaviors (Kheirbek et al., 2013; Bannerman et al., 2014). This functional segregation is underpinned not only by distinct anatomical connectivity but also by region-specific gene expression that is tightly coupled to physiological and functional properties of each domain (Thompson et al., 2008; Dong et al., 2009; Fanselow and Dong, 2010; Cembrowski et al., 2016). Defining the molecular determinants of this heterogeneity is therefore crucial for understanding hippocampal circuit formation and its disruption in neurological and psychiatric disorders.

Within the dentate gyrus (DG), the main gate of the hippocampal trisynaptic circuit, hilar mossy cells (MCs) constitute a unique and functionally pivotal population. These glutamatergic excitatory neurons extend long associational axons that bridge the dorsal and ventral portions of the DG, providing long-range connectivity across the hippocampus (Scharfman, 2016). MCs receive their primary inputs from granule cells (GCs), and in turn, project back onto both GCs (direct excitation) and local interneurons (indirect feedforward inhibition), thereby playing a crucial role in balancing excitation and inhibition in the DG (Scharfman and Myers, 2012). This dual control is considered essential for DG-dependent computation such as pattern separation and memory encoding (Jinde et al., 2012; Bui et al., 2018; Fredes et al., 2021; Li et al., 2022; Jeong et al., 2024a). By virtue of this connectivity, MCs are uniquely positioned to integrate and redistribute information across the hippocampal dorsoventral axis, shaping both cognitive and affective processing.

Dorsoventral heterogeneity within MCs has been documented in terms of their axonal projections (Botterill et al., 2021; Houser et al., 2021; Jeong et al., 2024b), dendritic morphology, intrinsic electrophysiology (Jinno et al., 2003), and synaptic connectivity (Fredes et al., 2021; Abdulmajeed et al., 2022). These anatomical and electrophysiological distinctions have, in turn, been linked to differential effects on the DG circuit function and behavior (Bauer et al., 2021; Fredes et al., 2021; Wang et al., 2021; Jeong et al., 2024a). Despite these well-defined cellular and functional distinctions, the genetic programs governing such heterogeneity remain poorly understood.

A central obstacle to addressing this question is their relatively low abundance among hippocampal principal cells, compounded by cellular heterogeneity within the hilus, which together make a cell-type–specific approach indispensable. To date, transcriptional profiling combined with fluorescence-based MC isolation has identified ∼59 candidate MC markers, a few of which were used to confirmed molecular heterogeneity against the Allen Brain Atlas In Situ Hybridization (ABA-ISH) database (Cembrowski et al., 2016). However, this work was focused almost exclusively on the dorsal DG region, so that a systematic dorsoventral comparison is still lacking. This knowledge gap limits our understanding of how MC support differential hippocampal functions and how their dysfunction might relate to distinct behavioral impairments.

To close this critical gap, we set out to systematically compare the molecular profiles of dorsal and ventral MC-enriched subpopulations in the DG. By employing translating ribosome affinity purification (TRAP) with *Drd2*-Cre-driven EGFP-L10a labeling, we captured the translatomes from dorsal and ventral hippocampal fractions enriched with MCs. This approach allowed us to uncover the distinct gene expression signatures that may underlie the functional heterogeneity of these populations. Our analysis revealed profoundly distinct translational signatures, with numerous genes enriched in either the dorsal or ventral fractions and predicted key upstream transcriptional regulators orchestrating these specific molecular profiles. Pathway analysis highlighted a significant functional segregation, where the dorsal population showed enrichment in genes vital for synaptic transmission and neuronal connectivity, while the ventral population was enriched in genes associated with energy metabolism and cellular maintenance. These findings provide a translatomic atlas for the dorsoventral heterogeneity within these hippocampal populations, offering crucial molecular insight into their differential contributions to circuit function in health and disease.

## Materials and Methods

### Animals

The Eef1a^LSL.EGFP-L10a/+^ transgenic (TG) mouse line (#030305, JAX stock) was imported from The Jackson Laboratory (JAX), and another TG mouse line, *Drd2*-Cre (#ER44, GENSAT), was obtained from GENSAT. All TG mice were bred with C57BL/6J mice (JAX) to obtain hemizygotes. Hemizygous *Drd2*-Cre mice were then cross-bred with hemizygous The Eef1a^LSL.EGFP-L10a/+^ mice to generate double transgenic animals. Animals were group-housed (2–5 per cage) under a 12 h light/dark cycle (7 AM to 7 PM) with standard chow (LabDiet) and water provided *ad libitum*. All experiments were conducted using age- (8–15 weeks) and sex-matched (male) littermates. All procedures were approved by the Animal Care and Use Committee of the Daegu Gyeongbuk Institute of Science and Technology (DGIST, IACUC #20011503-03).

### Immunohistochemistry

All animals were anesthetized via intraperitoneal injection of Avertin (250 mg/kg) and perfused transcardially with PBS, followed by 4% paraformaldehyde (PFA). The brains were extracted and incubated in 4% PFA overnight at 4°C before being transferred to 15% sucrose until they sank. Subsequently, they were transferred to 30% sucrose and incubated overnight at 4°C. The brains were coronally or horizontally sectioned into 40 µm slices using a cryostat (#CM3050S, Leica Biosystems). For immunostaining, each slice was incubated in blocking buffer (1× PBS, 0.2% BSA, 4% normal goat serum, 0.3% Triton X-100) at room temperature for 1 h. Following blocking, sections were incubated overnight at 4°C with primary antibodies diluted in the blocking buffer. The primary antibodies used were as follows: anti-NeuN (rabbit polyclonal, 1:1,000, #ABN78, Millipore), anti-GluR2/3 (rabbit polyclonal, 1:100, #ab1506, Millipore), anti-Satb1 (mouse monoclonal, 1:500, #sc-376096, Santa Cruz Biotechnology), and anti-calretinin (rabbit polyclonal, 1:500, #7699/3 h, SWANT). After incubation for 24 h, the sections were washed three times with washing buffer (1× PBS, 0.3% Triton X-100) for 10 min each. They were then incubated with either a Tyramide Signal Amplification (TSA) reagent (#B40955, Thermo Fisher Scientific) for anti-GluR2/3 or Alexa Fluor-conjugated secondary antibodies (1:400, Thermo Fisher Scientific) for other primary antibodies and counterstained with DRAQ5 (#62251, Thermo Fisher Scientific) at room temperature for 3 h. The sections were washed three times and mounted using ProLong Gold antifading mounting medium (#P36930, Thermo Fisher Scientific).

### In situ hybridization (ISH)

All chromogenic ISH images were obtained from the publicly available ABA-ISH database, including both coronal and sagittal sections. To compare spatial expression patterns with TRAP-seq (Fig. 2), we manually examined ABA-ISH images for each gene and assessed their expression in the dentate hilar region relative to other hippocampal subregions. For gene ontology-associated genes (Fig. 3), we evaluated whether these genes exhibited dorsoventral differences in hilar expression, focusing specifically on signal presence within the hilus rather than expression patterns in other hippocampal regions.

Fluorescence ISH was performed using the RNAscope Fluorescence Assay Kit (#323100, ACDbio) with Mm-Cyp26b1-C1 probe (#45421, ACDbio). Brains were fixed in 4% PFA overnight at 4°C, cryoprotected in 15% sucrose until sinking, followed by 30% sucrose overnight at 4°C, and sectioned coronally at 14 µm using a cryostat. Sections were mounted on Superfrost plus slides (#12-550-15, Thermo Fisher Scientific) and stored at −80°C. The RNAscope protocol was followed as per the manufacturer's instructions. Briefly, sections were fixed in prechilled 4% PFA for 15 min at 4°C, dehydrated in 50% and 70% and twice in 100% ethanol (5 min each), and incubated in hydrogen peroxide for 10 min at RT. Antigen retrieval was performed in boiling buffer for 5 min, followed by Protease III digestion for 30 min at 40°C. Sections were hybridized with probe (50 µl) for 2 h at 40°C and then rinsed in 1× wash buffer. Signal amplification was performed with Amp 1 (30 min), Amp 2 (30 min), and Amp 3 (15 min). Detection was carried out using Opal 520 dye (1:2,000 in TSA buffer, #FP1487001KT, Akoya Biosciences) for 30 min at 40°C. Slides were rinsed in 1× wash buffer between steps and counterstained with DAPI (#62248, Thermo Fisher Scientific).

### Image acquisition

Images were acquired using a Nikon A1 confocal microscope with a 20× objective lens under consistent imaging settings. Each image was collected as a *z*-stack at 4 µm intervals, tiled, and processed for brightness and contrast adjustment with NIS software (Nikon).

### Image quantification

All image analyses were performed using ImageJ (NIH). For counting MCs (labeled with EGFP-L10a) colocalized with markers (*Cyp26b1*, GluR2/3, and Satb1; Leranth et al., 1996; Hochgerner et al., 2018; Mundrucz et al., 2023), the hilar region was defined as the area between the upper and lower blades of the GC layer, excluding the subgranular zone (defined as two cell-body layers adjacent to the GC layer) and the portion of CA3 extending into the hilus. Positive signals were defined using the Color Threshold function. Threshold values were first determined using representative images and then applied consistently across all images to exclude background fluorescence. For quantifying fluorescence intensity (EGFP-L10a and *Cyp26b1*), individual cells were detected using the Analyze Particles function and defined as regions of interest (ROIs). Fluorescence intensity for each cell was measured as the integrated density, corresponding to the sum of pixel intensity values within each ROI. Signals below the defined fluorescence threshold were considered too weak for reliable detection and were excluded from analysis. All samples were processed and analyzed under identical imaging and analysis conditions.

### Translatomic profiling of dMCs and vMCs

#### Sample preparation and TRAP

TRAP assay was conducted with minor modifications to original procedure (Heiman et al., 2014), as described below. *Drd2*-Cre female mice (hemizygote) were cross-bred with Eef1a^LSL.EGFP-L10a/+^ male mice to produce MC-enriched TRAP (*Drd2*-Cre;Eef1a^LSL.EGFP-L10a/+^) mice. MC-enriched TRAP mice ∼10 weeks old were used for MC translatome analysis. After decapitation, the brains were rapidly soaked in the prechilled dissection buffer (1× HBSS, 2.5 mM HEPES, 35 mM glucose, 4 mM NaHCO_3_, 100 µg/ml cycloheximide, RNase-free water), pH 7.4, for 1 min. After incubation, the hippocampi from 25 mice were rapidly manually dissected and separated into dorsal and ventral parts on ice. Briefly, the brain was bisected along the midline to separate the hemispheres. Each hemisphere was then placed medial side up, and the diencephalon (thalamus and hypothalamus) was carefully removed with fine-tipped forceps to expose the hippocampus along the medial wall. The hippocampus was subsequently gently lifted and separated from the surrounding tissue using blunt-end forceps. Then each hippocampus was gently uncoiled along its curved structure and carefully extended into a linear configuration. Then, the medial portion of the ventral pole, corresponding to the anatomical location of the ventral subiculum based on gross hippocampal landmarks, was carefully removed under a microscope using fine-tipped forceps to minimize contamination (Extended Data Fig. 1-3). For dorsoventral subdivision, the hippocampus was separated according to proportional length, with the dorsal one-third defined as dorsal hippocampus and the remaining ventral two-thirds defined as the ventral hippocampus. This definition was guided by the known dorsoventral gradient of calretinin expression in MCs (Fujise et al., 1997; Jeong et al., 2024b), in which calretinin-positive MCs are predominantly located in the ventral DG and was applied consistently across samples. Five mice per assay were pooled for each TRAP to achieve the necessary minimum yield for RNA sequencing. Pooled hippocampal tissues were kept under dissection buffer for 30 min to minimize blood traces and then homogenized in ice-cold polysome extraction buffer [20 mM HEPES, 150 mM KCl, 10 mM MgCl_2_, 0.5 mM dithiothreitol, 100 µg/ml cycloheximide, protease inhibitors (EDTA-free), 400 unit/ml recombinant RNase inhibitors, and 200 unit/ml Superasin (#AM2696, Ambion)], pH 7.4, with a motor-driven Teflon glass homogenizer (#K8855100020, Thermo Fisher Scientific). Homogenates were cleared by centrifugation at 2,000 × *g* for 10 min at 4°C. NP-40 (#492018, EMD Biosciences) and 1,2-diheptanoyl-sn-glycero-3-phosphocholine (DHPC, #850306P, Avanti Polar Lipids) were added to the supernatant at a final concentration of 1% and 30 mM, respectively, followed by incubation on ice for 5 min. The clarified lysates were centrifuged for 10 min at 20,000 × *g* to separate insoluble material (cell debris) into a pellet, allowing the soluble fraction for downstream analysis. Monoclonal anti-EGFP antibodies (50 µg each of the clones 19C8 and 19F7; 100 µg total, corresponding to a final concentration of 100 µg/ml in 1 ml low-salt buffer) were immobilized onto Dyna magnetic beads (#123-21D, Invitrogen) via protein L (#PI-29997, Thermo Fisher Scientific). These EGFP-affinity beads were added to the supernatant, followed by incubation using an end-over-end rotator for 16 h at 4°C. Polysome-bound beads were washed three times in the high-salt washing buffer (20 mM HEPES, 350 mM KCl, 5 mM MgCl2, 1% NP-40, 0.5 mM dithiothreitol, 100 µg/ml cycloheximide, and RNase-free water), pH 7.4, with gentle agitation to resuspend the beads between washing steps. The beads were washed one more time with detergent-free washing buffer and immediately added to RLT buffer, followed by RNA purification with RNeasy Micro Kit (#74004, QIAGEN) with in-column DNase digestion. The quantity and integrity of purified RNA were determined by using a Quant-iT RiboGreen RNA Assay Kit (#R11490, Invitrogen) and Fragment Analyzer (AATI), which calculates an RNA quality number (RQN) ranging from 1 (highly degraded) to 10 (intact RNA). Only samples with an RQN greater than eight were selected for qPCR and RNA-seq.

#### qPCR analysis

The isolated RNA from the TRAP assay was used to synthesize cDNA with iScript cDNA Synthesis Kit (#1708891 Bio-Rad Laboratories). A 10 ng of cDNA was used for each quantitative real-time PCR (qPCR) reaction, and all samples were run in triplicate. qPCR analysis was carried out using SsoAdvanced Universal SYBR Green Supermix (#1725270, Bio-Rad) and AriaMx Real-Time PCR System (Agilent) following standard cycling conditions (95°C for 30 s, then 40 cycles of 95°C for 5 s and 60°C for 30 s). The relative quantitation of mRNA was calculated by the comparative Ct method after normalization to mouse *Map2*. The following primers were used: *Calb2*, 5′- TTTATGGAGGCTTGGCGGAA-3′ (forward) and 5′-TCATCATAGGGCCTGTTGGC-3′ (reverse); *Cyp26b1*, 5′-ATTCAGGAAGCGCAATGCAG-3′ (forward) and 5′-CGCCCCAGTAAGTGTGTCTT-3′ (reverse); and *Map2*, 5′-ATCTCTTCAGCACGACGGAC-3′ (forward) and 5′- CGTGAAGAGTAGCTTGGGGG-3′ (reverse). Nonspecific amplification was excluded by confirming a single peak in the melting curve analysis, which indicates a single and specific amplification product. To further validate the specificity, PCR products were also resolved on 2% agarose gels stained with ethidium bromide, and only samples showing a single band of the expected size were considered for analysis.

#### Data analysis

Full-length cDNA was generated from 500 pg of RNA with Ovation RNAseq v2 kit (NuGene). cDNA quality and quantity were checked on a Fragment Analyzer using DNA High Sensitivity Assay Kit (#DNF-486, AATI) prior to sequencing library preparation. Sequencing libraries were constructed by a TruSeq RNA library Prep Kit v2 (Illumina) and sequenced for 50 bp paired-end on Illumina HiSeq2500 using Rapid v2 sequencing chemistry (Illumina). For each library, the number of detected genes is presented in Extended Data Figure 3-1. Reads were then aligned to the mouse reference genome (GRCm38) using STAR v2.5.2b (Dobin et al., 2013). Read counts per gene were calculated using HTseq-count v0.7.2 (Anders et al., 2014). Differential expression analysis on raw read counts was performed in R using the edgeR package (Robinson et al., 2009). The package implements exact statistical methods based on generalized linear models. The particular feature of edgeR functionality is empirical Bayes methods that permit the estimation of gene-specific biological variation, even for experiments with minimal levels of biological replication. The quasi-likelihood method was implemented for differential expression analyses of bulk RNA-seq data. We first identified “expressed” genes as those with counts per million fragments mapped larger than 1 under at least two of the biological replicates. Finally, we identified differentially expressed genes (DEGs) as the ones that have the false discovery rate (FDR)-adjusted *p* values <0.05 and absolute log_2_-fold changes >0.58 (1.5-fold).

### Ingenuity pathway analysis (IPA)

To identify enriched canonical pathways and upstream regulatory elements between dorsal and ventral MCs, IPA (Qiagen) was performed using the DEG list described above. The analysis was based on the Ingenuity Knowledge Base (*Mus musculus*) as a reference. Canonical pathway analysis was used to evaluate the overrepresentation of DEGs in curated signaling pathways. The *z*-score algorithm was applied to predict the activation or inhibition state of each pathway based on the direction of gene expression changes (Krämer et al., 2014). Pathways with a |*z*-score| > 2 were considered to be significantly activated in either dorsal or ventral MCs.

### Statistical analysis

Statistical analyses were conducted using Prism 8 (GraphPad Software) and Microsoft Excel, and R-based bioinformatic tools. For translatomic data analysis, differential expression analysis was performed to directly compare dorsal and ventral samples using identical statistical thresholds. Genes were classified as dorsal- or ventral-subpopulation–enriched based on a minimum fold change (>1.5 ratio) and FDR-adjusted significance (*p* < 0.05) derived from this direct comparison. Gene Ontology (GO), Kyoto Encyclopedia of Genes and Genomes (KEGG), and canonical pathway analyses were performed using the DAVID functional annotation tool and IPA software, respectively. For all enrichment analyses, statistical significance was determined using Fisher's exact test (*p* < 0.05). Upstream regulator analysis was further conducted based on an activation *z*-score (|*z*| > 2) to predict the activation state of transcriptional regulators. For other experimental data, normality of the distribution was assessed using the Shapiro–Wilk test. Data meeting the assumptions of normality were analyzed using parametric tests, including Student's *t* test and two-way ANOVA followed by Tukey's post hoc test for multiple comparisons. All data are presented as mean ± standard error of the mean.

### Data and code availability

All RNA-seq data are publicly available in the GEO database under accession number GSE156004. All data in this paper is available upon request. This study does not generate any new code.

## Results

### Selective isolation of translating mRNA in *Drd2*-expressing populations enriched for MCs along the dorsoventral axis using TRAP

**Figure 1.**
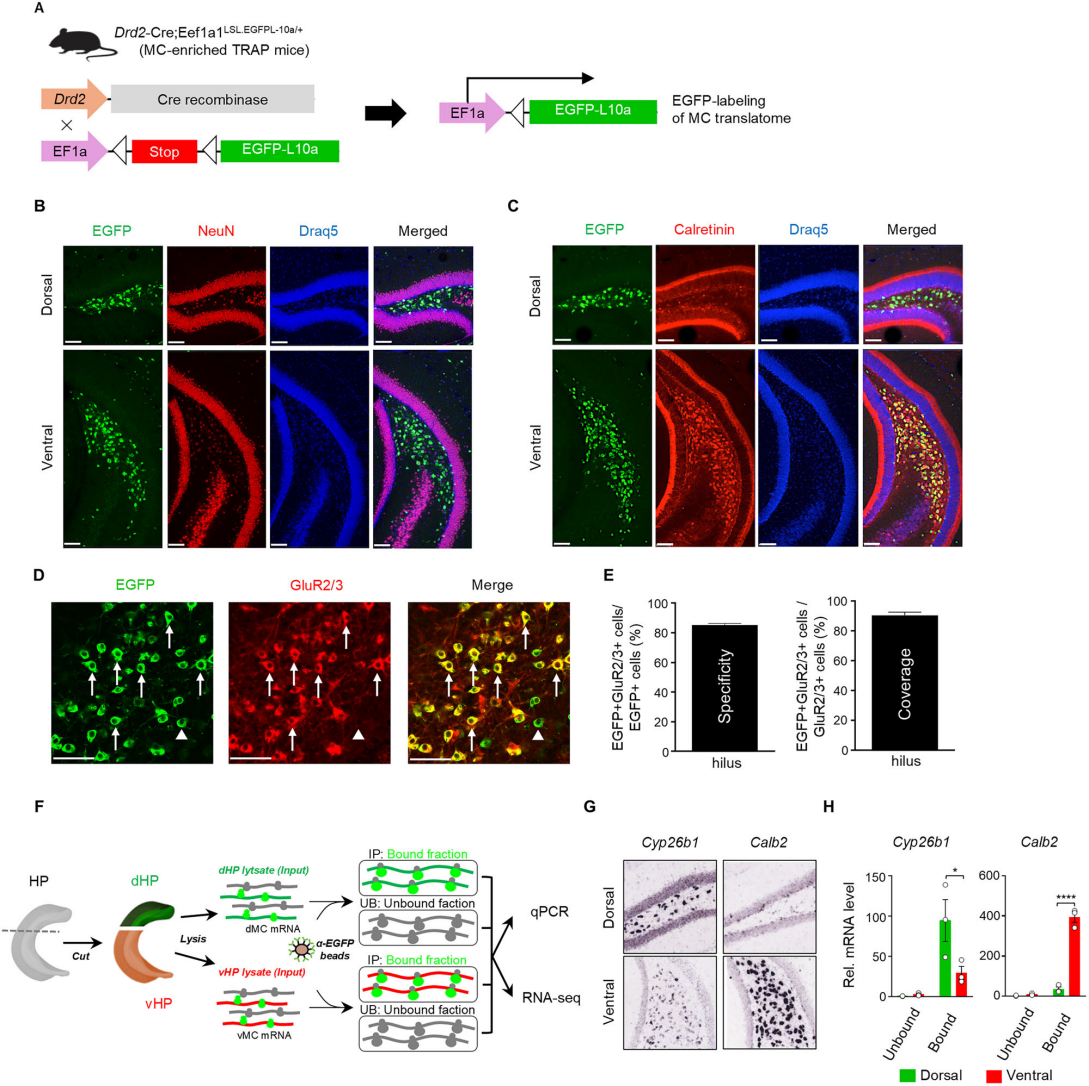
TRAP-based isolation of Drd2-expressing, MC-enriched population in the hippocampus. ***A***, Schematic of the genetic strategy. *Drd2*-Cre mice were crossed with Cre-dependent EGFP-L10a reporter mice to generate TRAP mice (*Drd2*-Cre;Eef1a1^LSL.EGFP-L10a/+^), enabling MC-specific expression of EGFP-tagged ribosomes. ***B, C***, Confocal images showing EGFP-L10a expression in the hilus along the dorsoventral axis of the DG. EGFP-L10a expressing neurons were colocalized with NeuN (neuronal marker) (***B***) and calretinin (ventral MC marker; ***C***). Scale bar, 100 µm. ***D***, Confocal images showing EGFP-L10a expressing neurons colocalized with GluR2/3 (pan-MC marker). Scale bar, 50 µm. ***E***, Quantification of specificity and coverage in EGFP-L10a expressing neurons colabeled with GluR2/3 MC marker along the dorsoventral axis of the DG. (*N* = 4–5, sections; *n* = 3, mice). ***F***, Schematic of the TRAP workflow. Dorsal and ventral hippocampal regions were microdissected and lysates were processed using anti-EGFP magnetic beads to isolate ribosome-bound mRNAs from MCs. ***G***, ABA-ISH images showing regional enrichment of dorsal (*Cyp26b1*) and ventral (*Calb2*) MC marker genes in the mouse hippocampus. For the dorsal enriched gene, *Cyp26b1*, experiment number, 79,568,022; probe, RP_071204_02_A12— coronal; sections, 24/47 (dorsal) and 31/47 (ventral). For the ventral enriched gene, *Calb2*, experiment number, 79,556,662; probe, RP_071204_01_B06—coronal, section, 29/59 (dorsal) and 38/59 (ventral). Scale bar, 100 µm. ***H***, qPCR analysis of TRAP (ribosome-bound) versus unbound mRNA samples from dorsal and ventral regions, validating dorsal enrichment of *Cyp26b1* and ventral enrichment of *Calb2* (*n* = 3, pooled hippocampal tissues). Two-way ANOVA revealed significant differences for *Cyp26b1* (unbound vs bound, *F*_(1,8)_ = 19.61; *p* = 0.0022; dorsoventral axis, *F*_(1,8)_ = 5.339; *p* = 0.0496; interaction, *F*_(1,8)_ = 6.048; *p* = 0.0394) and *Calb2* (unbound vs bound, *F*_(1,8)_ = 508.5; *p* < 0.0001; dorsoventral axis, *F*_(1,8)_ = 159.4; *p* < 0.0001; interaction, *F*_(1,8)_ = 147.3; *p* < 0.0001). Post hoc analysis was performed using Tukey's multiple-comparison test (∗*p* < 0.05; ∗∗∗∗*p* < 0.0001). See also Extended Data Figures 1-1–1-4.

Accurately analyzing specific molecular characteristics of hilar MCs is challenging due to the diverse cell types in the dentate hilus. To overcome this, we employed TRAP, a method that enables cell-type–specific enrichment of actively translating mRNAs by immunoprecipitating EGFP-tagged L10a ribosomal subunits (EGFP-L10a) from genetically defined cell populations (Heiman et al., 2008, 2014). While *Calcrl*-Cre and *Drd2*-Cre lines were previously reported to exhibit Cre activity in MCs (Gangarossa et al., 2012; Jinde et al., 2012), the *Calcrl*-Cre line shows limited specificity in double transgenic animals likely due to transient developmental Cre activity in CA3 pyramidal cells. In contrast, the *Drd2*-Cre line exhibits robust Cre recombinase activity in MCs of the DG while also labeling a minor subset of other *Drd2*-expressing hippocampal cell populations (Gangarossa et al., 2012; Puighermanal et al., 2015). Accordingly, we generated *Drd2*-Cre;Eef1a1^LSL.EGFP-L10a/+^ (MC-enriched TRAP) mice to isolate ribosome-bound mRNAs from these MC-enriched populations via the EGFP-tagged ribosomal L10a subunit (Fig. 1*A*).

In MC-enriched TRAP mice, EGFP-L10a expression was observed predominantly in the dentate hilar region along the entire dorsoventral axis (Fig. 1*B*). Immunostaining confirmed that EGFP-L10a expression colocalized with calretinin (*Calb2*), a known marker primarily expressed in ventral MCs (Fig. 1*C*; Fujise et al., 1997). However, as calretinin does not label all MCs (especially in the dorsal region) and the hilus contains other cell types, we further verified MC specificity across the entire dorsoventral axis. Immunostaining for GluR2/3, a well-established MC marker (Leranth et al., 1996), across the dorsoventral axis of the DG (Extended Data Fig. 1-1) revealed high specificity (85 ± 1.9% of EGFP-L10a–expressing neurons were GluR2/3-positive) and substantial MC coverage (90 ± 3.7% of GluR2/3-positive MCs were labeled by EGFP-L10a; Fig. 1*D*,*E*). We quantified the distribution of EGFP-L10a–expressing neurons across the entire hippocampal formation. However, EGFP-L10a signal was also observed in sparse GABAergic interneurons at other CA subregions, as well as in the neurons within the ventral subiculum (Extended Data Fig. 1-2*A*), which reproduce the known expression profile of *Drd2*-Cre mice (Puighermanal et al., 2015; Extended Data Fig. 1-2*D*,*E*). Furthermore, total fluorescence intensity analysis confirmed that the hilus accounted for a markedly higher EGFP-L10a signal (over 65%) compared with other regions (Extended Data Fig. 1-2*F*,*G*). While acknowledging the presence of off-target *Drd2*-expressing populations, our histological quantification demonstrates that the captured population is heavily enriched for MCs, validating the feasibility of characterizing these translational profiles using our TRAP approach.

10.1523/ENEURO.0236-25.2026.f1-1 Figure 1-1 Selective expression of EGFP-L10a in MCs across the dorsoventral axis of the DG in MC-enriched TRAP mice. (A) Representative horizontal sections from *Drd2*-TRAP mice show selective expression of the EGFP-L10 in MCs along the dorsoventral axis of the DG. Immunohistochemistry for GluR2/3 (red), a marker for MCs, confirms colocalization with EGFP-L10a in the hilar region. White dashed lines outline the hilus. Scale bars, 100 μm. Download Figure 1-1, TIF file.

**Figure 2.**
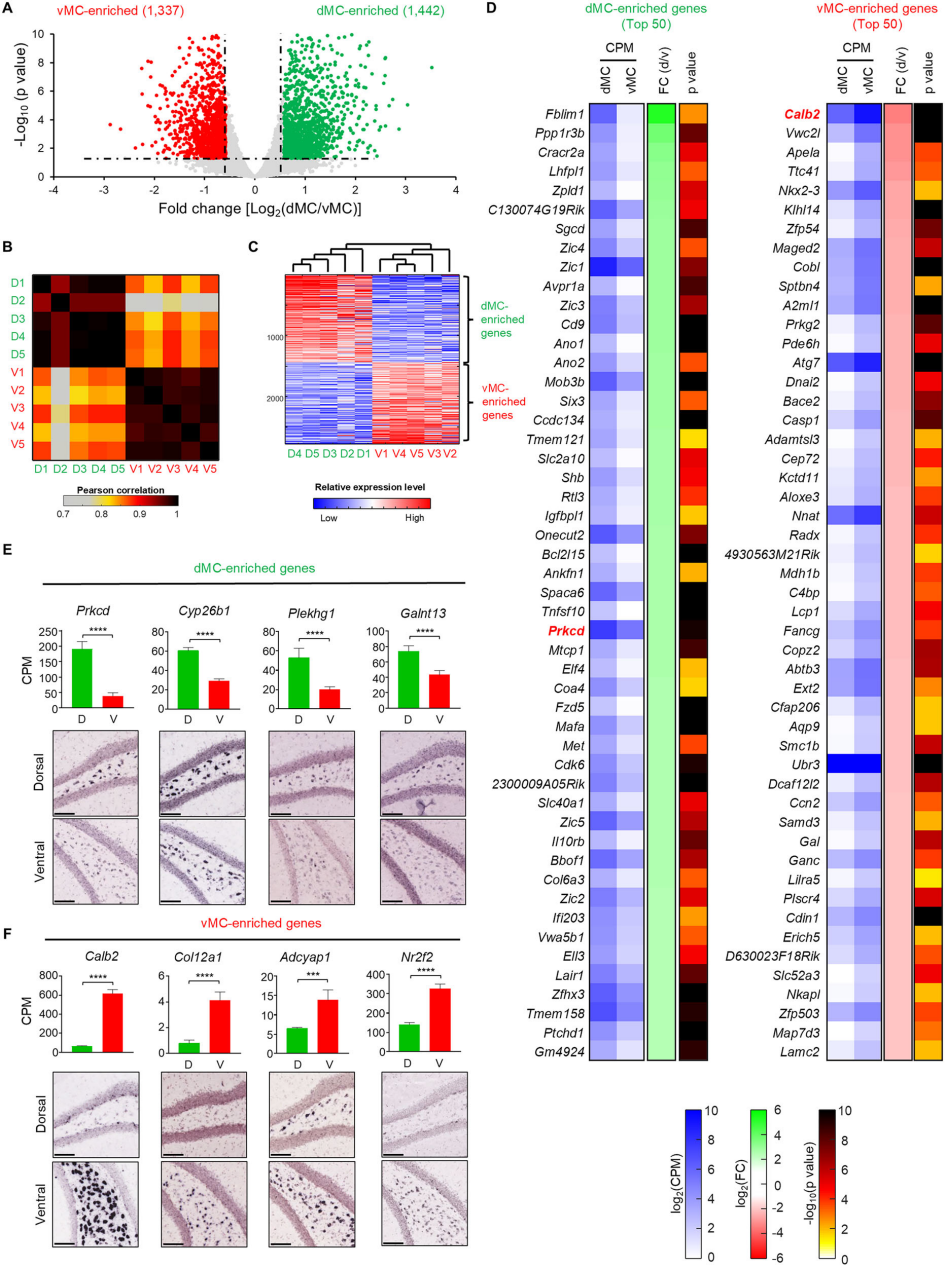
*Drd2*-driven translatomic profiling reveals DEGs between dorsal and ventral MC-enriched populations. ***A***, Volcano plot displaying DEGs between dorsal and ventral MC-enriched samples (the dash line indicates cutoff: FDR-adjusted *p* value <0.05, |log_2_FC| > 0.58). Genes enriched in each subpopulation were plotted in green or red dots, as indicated. ***B***, MDS plot showing distinct clustering of TRAP-based translatome from five dorsal and five ventral fractions groups (*n* = 5 mice per assay), indicating clear segregation based on gene expression profiles. ***C***, Heatmap of DEGs between dorsal and ventral MC-enriched samples, generated via hierarchical clustering samples based on normalized expression values. ***D***, Heatmap showing the top 50 DEGs with the highest expression enrichment in dorsal (left) and ventral (right) MC-enriched samples. ***E, F***, ABA-ISH images showing DEGs enriched in the dorsal or ventral hilus. Representative dorsal enriched genes: *Prkcd*, *Cyp26b1*, *Plekhg1*, and *Galnt13* (***E***). Representative ventral enriched genes: *Calb2*, *Col12a1*, *Adcyap1*, and *Nr2f2* (***E***). (FDR-adjusted *p < 0.05; ***p < 0.001; ****p < 0.0001.) For the dorsal enriched genes, *Prkcd*, experiment number, 70,301,274; probe, RP_050309_02_B09, coronal, sections, 54/114 (dorsal) and 68/115 (ventral); *Cyp26b1*, experiment number, 79,568,022; probe, RP_071204_02_A12, coronal, sections, 24/47 (dorsal) and 30/47 (ventral); *Plekhg1*, experiment number, 71,670,687; probe, RP_050505_04_G05, coronal; sections, 23/58 (dorsal) and 35/58 (ventral); *Galnt13*, experiment number, 72,472,803; probe, RP_051101_02_F07, coronal; sections, 20/49 (dorsal) and 26/49 (ventral). For the ventral enriched genes, *Calb2*, experiment number, 79,556,662; probe, RP_071204_01_B06, coronal; section, 29/59 (dorsal) and 38/59 (ventral); *Col12a1*, experiment, 73,817,431; probe, RP_051101_01_A06, coronal, sections, 54/115 (dorsal) and 67/115 (ventral); *Adcyap1*, experiment, 74,511,882; probe, RP_051121_04_A03, coronal, sections, 57/119 (dorsal) and 70/119 (ventral); *Nr2f2*, experiment, 112,646,890; probe, RP_110407_01_A05, coronal, sections, 22/50 (dorsal) and 29/50 (ventral). Scale bar, 100 µm. See also Extended Data Figures 2-1 and 2-2.

10.1523/ENEURO.0236-25.2026.f1-2 Figure 1-2 EGFP-L10a expressions along the dorsoventral axis of the hippocampus of MC-enriched TRAP mice. (A) Representative fluorescence images showing the distribution of EGFP-L10a (green) and NeuN (red) in the dorsal (top row) and ventral (bottom row) hippocampus. In both regions, strong EGFP-L10a expression is observed in MCs in the hilus. Sparse expression is also detected in interneurons located in CA1 so, sr, and slm. (B and C) Single-cell EGFP-L10a fluorescent intensity in the dorsal (B) and ventral (C) hippocampal subregions. Fluorescent intensity was calculated as pixel area multiplied by intensity per cell. (D and E) Pie chart showing the proportional distribution of EGFP-L10a positive cell number across the dorsal (D) and ventral (E) hippocampal subregions. For the dorsal hippocampus. (F and G) Pie chart showing total EGFP-L10a fluorescent intensity in each hippocampal subregion of the dorsal (F) and ventral (G) hippocampus. CA1–3, Cornu Ammonis areas; DG, dentate gyrus; GCL, granule cell layer; hil, hilus; mo, molecular layer; slm, stratum lacunosum-moleculare; sr, stratum radiatum; so, stratum oriens; DS, dorsal subiculum; VS, ventral subiculum. Scale bars, 100 µm. Download Figure 1-2, TIF file.

**Figure 3.**
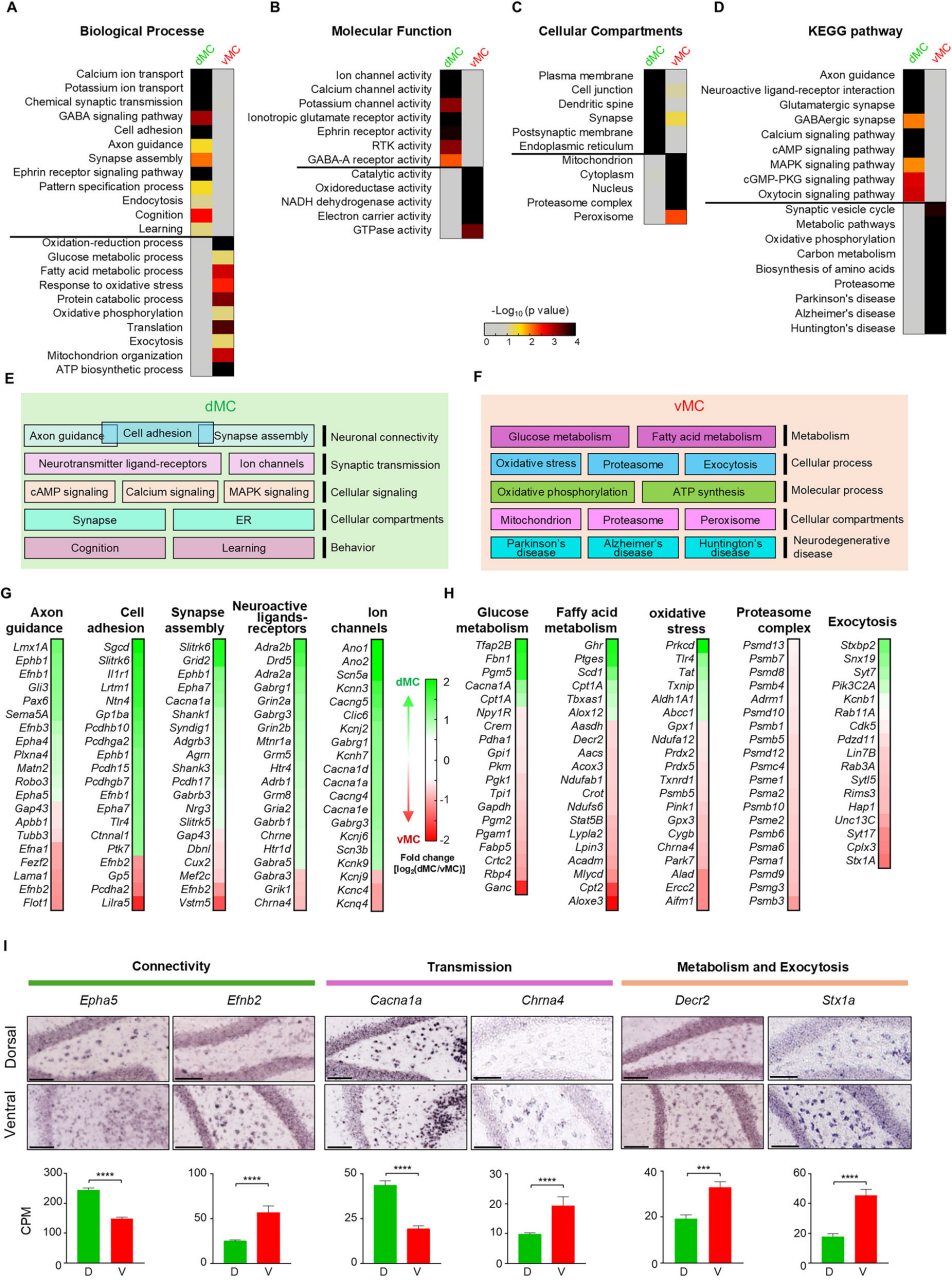
Distinct neurobiological properties between dorsal and ventral MC-enriched populations. ***A–D***, GO and KEGG pathway enrichment analyses of DEGs in dorsal and ventral MC-enriched populations. Functional categories include biological processes (***A***), molecular functions (***B***), cellular components (***C***), and signaling pathways (***D***). Significance was determined using Fisher's exact test (*p* < 0.05). ***E, F***, Graphical representations of dorsal MC- (***E***) or ventral MC-enriched (***F***) gene families with five layers of functional categories that organize neuronal properties. ***G*, *H***, Heatmaps showing the top 20 DEGs exhibiting the highest fold changes between dorsal (***G***) and ventral MC-enriched populations (***H***) in each functional category. ***I***, ABA-ISH images showing DEGs enriched in the dorsal or ventral hilus (FDR-adjusted *p < 0.05; ***p < 0.001; ****p < 0.0001). For the DEGs, *Epha5*, experiment, 71,587,625; probe, RP_050915_04_F05, sagittal, sections, 7/18 (dorsal) and 7/18 (ventral); *Efnb2*, experiment, 72,079,959; probe, RP_051017_03_B08, coronal, sections, 28/60 (dorsal) and 37/60 (ventral); *Cacna1a*, experiment, 79,761,029; RP_071204_04_A01, sagittal, sections, 13/18 (dorsal) and 13/18; *Chrna4*, experiment, 1,173; probe, RP_Baylor_102618, coronal, sections, 29/56 (dorsal) and 35/56 (ventral); *Decr2*, experiment, 71,924,363; probe, RP_050614_04_D05, coronal, sections, 27/57 (dorsal) and 35/57 (ventral); *Stx1a*, experiment, 2,645; probe, RP_Baylor_253979, coronal; sections, 30/56 (dorsal) and 36/56 (ventral). Scale bar, 100 µm. See also Extended Data Figure 3-1.

10.1523/ENEURO.0236-25.2026.f1-3 Figure 1-3 Anatomical validation of ventral subiculum microdissection. (A) Schematic illustration of hippocampal anatomy showing the location of the subiculum relative to adjacent hippocampal subregions. (B) Representative image of microdissection of the ventral subiculum from the hippocampus performed under a dissecting microscope. The ventral subiculum was anatomically identified and carefully isolated to minimize contamination from adjacent hippocampal regions. (C) Representative longitudinal hippocampal sections along the dorsoventral axis. dHIP, dorsal hippocampus; vHIP, ventral hippocampus; CA1–3, Cornu Ammonis areas; DG, dentate gyrus; Sub, Subiculum; DS, dorsal subiculum; VS, ventral subiculum; PrS, presubiculum. Scale bars, 200 µm. Download Figure 1-3, TIF file.

**Figure 4.**
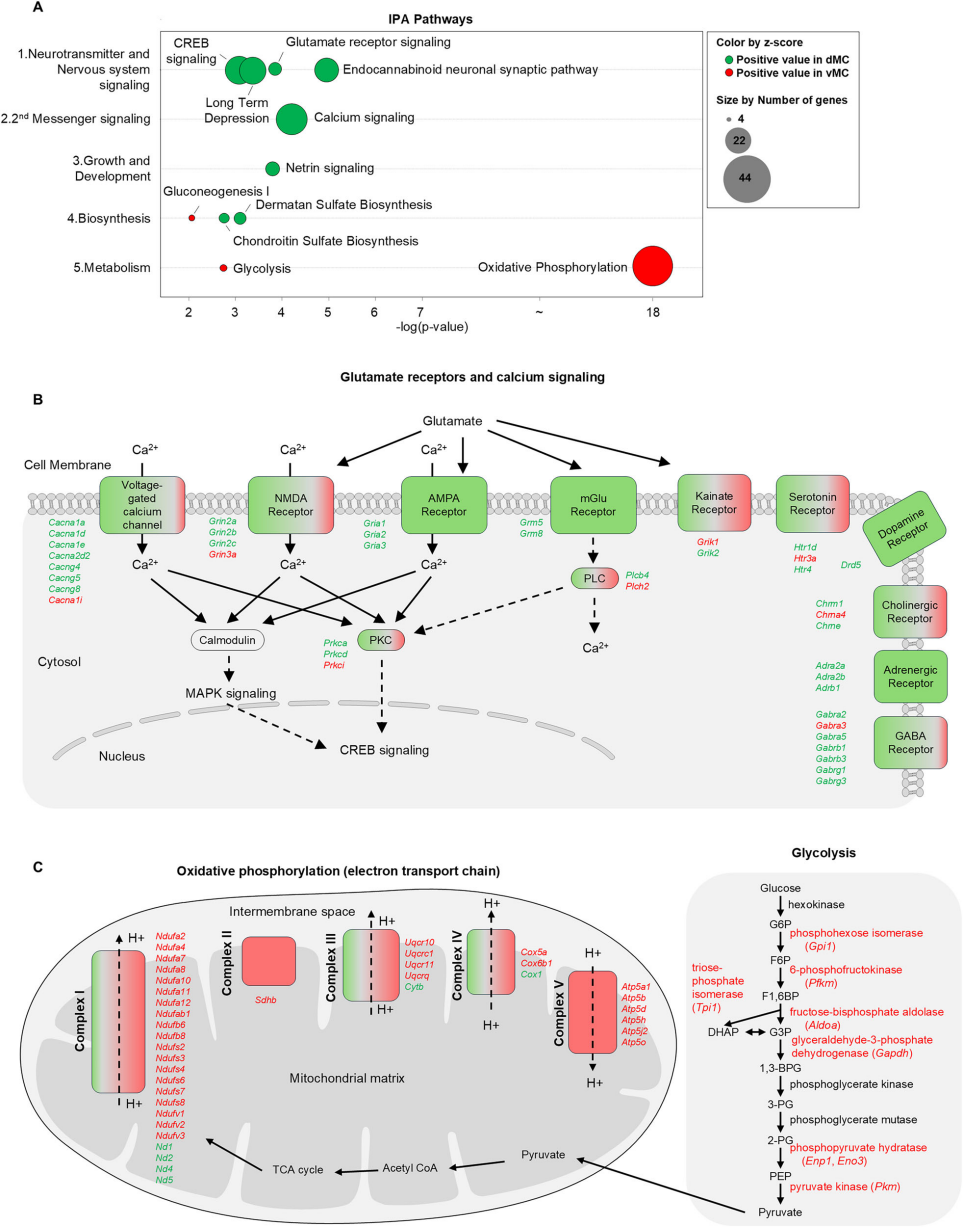
Dorsal- and ventral MC-enriched populations exhibit distinct canonical pathways. ***A***, Bubble plot showing canonical pathways identified through IPA of DEGs between dorsal and ventral MC-enriched populations. Statistical significance was determined using Fisher's exact test (*p* < 0.05). The color of each bubble represents the activation *z*-score, indicating predicted activation in dorsal (positive *z*-score) or ventral (negative *z*-score) MC-enriched populations. ***B***, Schematic of the ion channels, neuroactive receptors, and calcium signaling pathway, showing predicted activation in dorsal MCs. Green denotes enrichment in dorsal MC-enriched population; red denotes enrichment in ventral ones. ***C***, Diagram of glycolysis and oxidative phosphorylation pathway, showing predicted activation in ventral MCs. Green denotes enrichment in dorsal MC-enriched population; red denotes enrichment in ventral ones. See also Extended Data Figures 4-1–4-3.

10.1523/ENEURO.0236-25.2026.f1-4 Figure 1-4 Histological validation of *Cyp26b1* gene as a dorsal MC marker. (A) Representative images showing selective expression of *Cyp26b1* gene in MCs along the dorsoventral axis of the DG. *In situ* hybridization (ISH) for *Cyp26b1* mRNA (green) was combined with immunohistochemistry for the MC marker Satb1 protein (red) in the dorsal and ventral hippocampus. Arrowheads indicate *Cyp26b1 ISH* signals colocalized with Satb1 immunoreactivity in the hilus (outlined by white dashed lines). (B and C) Quantification of MC specificity (B) and coverage (C) of *Cyp26b1*expressing neurons colocalized with Satb1 along the dorsoventral axis of the DG (n = 3 mice). (Unpaired two-tailed Student’s t-test, ***p < 0.001). (C) Quantification of individual *Cyp26b1* fluorescent intensity in the dorsal and ventral DG (Unpaired two-tailed Student’s t-test, ***p < 0.0001). Cells were identified and quantified using threshold-based detection of fluorescent signals. Fluorescent intensity was calculated as the product of the pixel area and mean intensity per cell. Scale bars, 100 µm. Download Figure 1-4, TIF file.

Dorsal and ventral MCs exhibit distinct axonal projection patterns along the dorsoventral axis, which closely correspond to calretinin-negative and calretinin-positive MC populations, respectively (Botterill et al., 2021; Houser et al., 2021; Jeong et al., 2024b). Consistent with this distinction, calretinin expression exhibits a discrete distribution pattern along the dorsoventral axis. It is absent in the dorsal one-third of the DG, followed by a narrow zone with intermingled calretinin-negative and calretinin-positive expression and is robustly expressed in the ventral two-thirds (Jeong et al., 2024b). This anatomical and molecular boundary provides a reliable criterion for defining dorsal and ventral MC subpopulations. Based on this, we microdissected the hippocampi of TRAP mice into dorsal (one-third) and ventral (two-thirds) segments while carefully excluding with the ventral subiculum (Extended Data Fig. 1-3). Then, EGFP-L10a-bound polysomes were then immunoprecipitated from these dorsal and ventral homogenates using anti-EGFP antibodies (Fig. 1*F*). To confirm MC transcript enrichment and dorsal/ventral profile separation, we first verified distinct dorsal and ventral MC markers. *Calb2*, a well-characterized ventral MC marker, was used directly, whereas *Cyp26b1* has been reported as a putative dorsal MC marker (Hochgerner et al., 2018). We further validated its MC specificity using ISH for *Cyp26b1* combined with Satb1 immunohistochemistry, a known MC marker (Mundrucz et al., 2023). The majority of *Cyp26b1* expressing neurons colocalized with Satb1 and were significantly enriched in dorsal MCs (Extended Data Fig. 1-4). Using these dorsal and ventral MC markers, we performed qPCR on both the immunoprecipitated (ribosome-bound) and supernatant (ribosome-unbound) fractions. The qPCR analysis showed significant enrichment of both markers in the ribosome-bound fraction compared with the unbound fraction (Fig. 1*G*,*H*). Specifically, the dorsal-bound samples showed enrichment for *Cyp26b1*, while the ventral-bound samples were enriched for *Calb2*, whose expression patterns were also confirmed from the ABA-ISH database (Fig. 1*G*,*H*). These findings validate that our TRAP procedure successfully enriches for actively translating MC transcripts and effectively captures distinct molecular profiles of dorsal and ventral MC subpopulations.

### Differential gene expressions between dorsal and ventral populations

High-throughput RNA sequencing of TRAP-enriched MC translatome from dorsal and ventral hippocampal regions defined their molecular profiles (Fig. 1*F*). Next, using the edgeR pipeline with a stringent FDR criterion (FDR-adjusted *p* value <0.05), we identified DEGs between the dorsal and ventral TRAP samples. This analysis revealed 1,442 genes significantly enriched in dorsal samples and 1,337 genes in ventral samples, highlighting distinct translatomic profiles in these subpopulations (Fig. 2*A*). Multidimensional scaling (MDS) and hierarchical clustering based on the identified DEGs further confirmed clear separation and robust clustering of the dorsal versus ventral TRAP samples, demonstrating reproducibility across biological replicates (Fig. 2*B*,*C*).

10.1523/ENEURO.0236-25.2026.f2-1 Figure 2-1 Quantitative TRAP-seq validation of ventral subiculum marker genes. (A) Representative *in situ* hybridization images showing expression patterns of ventral subiculum marker genes (Grp, Dlk1, Gpc3) in the dorsal and ventral hippocampus. (B) Quantification of marker gene expression in dorsal (green) and ventral (red) TRAP-seq datasets. Notably, transcript levels of these ventral subiculum markers are detected low in both dorsal or ventral TRAP-seq datasets and even *Gpc3* seems de enriched in ventral dataset as compared to dorsal one (FDR-adjusted p > 0.05). Grp, experiment: 1363, probe: RP_Baylor_103371 – coronal, sections: 29/55 (dorsal) and 35/55 (ventral); Dlk1, experiment: 71587885, probe: RP_050725_03_A10 – coronal, sections: 56/117 (dorsal) and 71/117 (ventral); Gpc3, experiment: 71020431, probe: RP_050329_01_C10 – coronal, sections: 51/111 (dorsal) and 65/111 (ventral). PoDG, polymorphic layer of the dentate gyrus; DS, dorsal subiculum; VS, ventral subiculum; DG, dentate gyrus; CA1–3, Cornu Ammonis areas. Scale bars, 600 µm. Download Figure 2-1, TIF file.

10.1523/ENEURO.0236-25.2026.f2-2 Figure 2-2 Comparable expressions of GABAergic interneuron markers between dorsal and ventral MC-enriched populations (A) Volcano plot showing that GABAergic interneuron marker genes are not significantly enrichment between dorsal and ventral MC-enriched samples. Red and green dots represent genes enriched in ventral and dorsal MC-enriched samples, respectively, while gray dots indicate non-differentially expressed genes. Blue labels highlight GABAergic interneuron markers (*Gad1*, *Gad2*, and *Slc32a1*), which showed no significant differences between dorsal and ventral MC-enriched samples. Download Figure 2-2, TIF file.

Crucially, TRAP-seq analysis revealed no significant enrichment of ventral subiculum-specific markers (Cembrowski et al., 2018) in dataset (Extended Data Fig. 2-1), indicating minimal contamination from ventral subiculum tissue during microdissection. We also performed an internal comparative analysis to assess potential contamination bias from off-target hippocampal GABAergic neurons. Canonical interneuron markers (*Gad1*, *Gad2*, *Slc32a1*) showed comparable expression levels between dorsal and ventral samples (Extended Data Fig. 2-2), demonstrating that off-target GABAergic interneuron-derived signals were balanced across samples and did not drive the observed dorsoventral differences.

Top-ranked DEGs included *Fblim1* (encoding the focal adhesion-associated adaptor protein Migfilin), which was enriched in dorsal MCs, and *Calb2* (encoding the calcium-binding protein calretinin), which is consistent with its known ventral MC preference (Fig. 2*D*). To validate these differential expression patterns, we cross-validated DEGs with ABA-ISH images. We manually examined ISH signals for genes exhibiting hilar-specific and dorsoventral differential expression based on visual inspection. We found that dorsal enriched genes (*Prkcd*, *Cyp26b1*, *Plekhg1*, and *Galnt13*) showed stronger signals in the dorsal hilus (Fig. 2*E*), whereas ventral enriched genes (*Calb2*, *Col12a1*, *Adcyap1*, and *Nr2f2*) displayed higher expression in the ventral hilus (Fig. 2*F*). Among these, *Cyp26b1*, *Calb2*, and *Adcyap1* were consistent with previously reported MC markers (Fujise et al., 1997; Hochgerner et al., 2018; Zhang et al., 2021; Oh et al., 2026). Collectively, these analyses strongly suggest that dorsal and ventral MC subpopulations possess distinct molecular signatures.

### Distinct neurobiological properties along the dorsoventral axis

GO-based functional annotation analysis of DEG lists using the DAVID bioinformatics resource identified key biological processes in each MC subpopulation (Extended Data Fig. 3-1). Based on the “Biological Process” and “Molecular Function” categories, dorsal enriched genes were predominantly associated with axonal guidance, cell adhesion, synapse assembly, and synaptic transmission. In contrast, ventral enriched genes showed higher representation in categories related to metabolic processes, proteasomal protein catabolism, and synaptic vesicle recycling (Fig. 3*A*,*B*). Analysis of the “Cellular Compartment” category further supported this dichotomy, where dorsal enriched genes were associated with the plasma membrane and synapse, while ventral enriched genes were linked to the mitochondrion and proteasome complex (Fig. 3*C*,*D*). To synthesize these findings, we reclassified the enriched GO terms and associated DEGs into functionally relevant groups: neuronal connectivity (axon guidance, cell adhesion, synapse assembly), synaptic transmission (neuroactive ligand–receptor interactions, ion channels), neuronal metabolism (glucose and fatty acid metabolism), and cellular processes (oxidative stress response, proteasome function, exocytosis; Fig. 3*E*,*F*). Individual genes within these functional categories were ranked by absolute fold enrichment between the subpopulations (Fig. 3*E*,*H*).

10.1523/ENEURO.0236-25.2026.f3-1 Figure 3-1 DEGs between dorsal and ventral MC-enriched populations and their functional enrichment analysis Download Figure 3-1, XLS file.

Beyond GO analysis, direct comparison with ABA-ISH images further validated differential expressions of specific genes (Fig. 3*I*). Notably, distinct axon guidance gene subsets, including Eph-ephrin family members such as *Epha5* and *Efnb2*, were differentially expressed between dorsal and ventral MCs. Furthermore, *Cacna1a*, encoding a subunit of voltage-dependent P/Q-type calcium channels, was enriched in dorsal MCs, while *Chrna4*, encoding a subunit of nicotinic acetylcholine receptors, was predominantly expressed in ventral MCs. Consistent with the GO analysis, the metabolic gene *Decr2* and the exocytosis-related gene *Stx1a* were enriched in ventral MCs. Altogether, our genomic approach revealed distinct neurobiological properties between the dorsal and ventral populations enriched for MCs.

### Pathway network analysis reveals distinct signaling and metabolic profiles

IPA software further explored DEG-associated functional pathways (Extended Data Fig. 4-2), revealing significant differences in canonical pathway enrichment between dorsal and ventral MCs (Fig. 4*A*). The dorsal dataset showed significant enrichment of glycosaminoglycan/proteoglycan biosynthesis pathways, including “Dermatan Sulfate Biosynthesis” and “Chondroitin Sulfate Biosynthesis” (*z* > 3), which produce glycosaminoglycan chains forming proteoglycans implicated in axonal guidance and patterning (Brittis et al., 1992; Sugahara and Mikami, 2007). Additionally, the “Calcium Signaling” pathway was significantly enriched in the dorsal population (*z* > 2), suggesting potential differences in excitability (Fig. 4*A*). Conversely, ventral MCs exhibited strong enrichment in metabolic pathways, particularly “Oxidative Phosphorylation” (*z* < −4) and “Glycolysis” (*z* < −2), with significant upregulation of numerous genes encoding components of the electron transport chain and glycolytic enzymes, indicating elevated metabolic activity (Fig. 4*A*).

10.1523/ENEURO.0236-25.2026.f4-1 Figure 4-1 Distribution of Neurotransmitter receptors and Ion channels in MC-enriched population. (A and B) Heatmaps showing the expression pattern of DEGs in neuroactive receptors (A) and ion channels (B) in dorsal and ventral MCs. Functional categories for each gene are indicated. Raw expression levels are presented as log₂-transformed counts per million (log_2_[CPM]). Adjacent heatmaps display the corresponding log₂ fold changes (log_2_[FC]) and adjusted p-values (log_10_[adjusted p-value] for each gene, using distinct color gradients. Download Figure 4-1, TIF file.

10.1523/ENEURO.0236-25.2026.f4-2 Figure 4-2 Canonical pathway analysis and predicted upstream transcriptional regulators of dorsal and ventral MC-enriched populations Download Figure 4-2, XLS file.

10.1523/ENEURO.0236-25.2026.f4-3 Figure 4-3 List of gene expressions related to neurotransmitter receptors and ion channels in dorsal and ventral MC-enriched populations Download Figure 4-3, XLS file.

Based on the convergence between IPA and DAVID analyses, we examined the “Synaptic Transmission” and “Energy Metabolism” pathways in more detail (Fig. 4*B*,*C*). Consistent with enrichment in calcium signaling, the dorsal population showed increased expression of multiple voltage-gated calcium channel subunit genes (*Cacna1a*, *Cacna1d*, *Cacna1e*, *Cacna2d2*, *Cacng4*, *Cacng5*, *Cacng8*) and glutamate receptor subunit genes (*Gria1*, *Gria2*, *Gria3*, *Grin2a*, *Grin2b*, *Grin2c*, *Grm5*, *Grm8*; Fig. 4*B*; Extended Data Figs. 4-1, 4-3). Conversely, the ventral dataset displayed significant upregulation of nearly all core glycolytic enzyme genes (7 genes) and a large cohort of genes involved in oxidative phosphorylation (40 genes), including components of mitochondrial respiratory chain complexes I–IV (Fig. 4*C*). Collectively, these results demonstrate distinct molecular signatures in each subpopulation, providing robust evidence for molecular heterogeneity along the dorsoventral axis of the DG.

### Prediction of distinct upstream transcriptional regulators

IPA Upstream Regulator Analysis predicted transcription factors potentially driving the molecular heterogeneity between these subpopulations, revealing distinct sets of candidate upstream regulators (Table 1, Extended Data Fig. 4-2). In dorsal dataset, key master regulators such as Tcf7L2, Egr2, Sox2, and Sox10 were predicted to be activated. In contrast, Ppargc1a, Smad7, Nfe2l2, and Rb1 were predicted as major upstream regulators shaping the ventral translatome. Several of these predicted regulators align with observed molecular profiles. For instance, Tcf7L2, a key component of the Wnt signaling pathway, is linked to neuronal excitability and seizure susceptibility (Hodges and Lugo, 2018; Medina et al., 2018), potentially influencing the synaptic and intrinsic properties of the dorsal population. Conversely, Ppargc1a, a master regulator of mitochondrial biogenesis and oxidative metabolism, coactivates NRF1 and NRF2, promoting gene expression involved in oxidative phosphorylation and electron transport chain function to support neuronal ATP production and energy homeostasis (Wu et al., 1999; St-Pierre et al., 2006). Collectively, these predictions suggest that distinct transcriptional regulatory networks operate in dorsal and ventral populations, likely orchestrating the expression programs tightly coupled to their functional roles along the longitudinal axis of the DG.

**Table 1.** Prediction of master gene regulators in dorsal and ventral MC-enriched populations

Activation state	Upstream regulator	Description	Activation *z*-score	*p* value of overlap
Activated in dMC	Tcf7L2	Transcription factor 7 like 2, T-cell specific, HMG box	4.743	0.0000311
	Egr2	Early growth response 2	3.312	0.002
	Sox2	SRY (sex determining region Y)-box 2	3.031	0.000464
	Sox10	SRY (sex determining region Y)-box 10	2.559	0.00041
Activated in vMC	Ppargc1A	Peroxisome proliferative activated receptor, gamma, coactivator 1 alpha	−2.169	0.00209
	Smad7	SMAD family member 7	−2.393	0.00946
	Nfe2L2	Nuclear factor, erythroid derived 2, like 2	−2.904	0.000172
	Rb1	RB transcriptional corepressor 1	−3.601	0.000000716

Master gene regulators are differentially activated in dorsal versus ventral MC-enriched populations, based on differential translatomic profiling. Regulators are grouped by predicted activation state and sorted by activation *z*-score. Positive *z*-scores indicate activation in dorsal MCs; negative *z*-scores indicate activation in ventral MCs.

## Discussion

Molecular profiling frequently provides critical insights underlying functional diversity within populations that appears homogeneous populations. Although the molecular landscapes of the principal hippocampal neurons (CA3, CA1, GC) along the dorsoventral axis have been well characterized, MCs in the DG remain comparatively underexplored. The HippoSeq study by Cembrowski et al. (2016) provided the first transcriptomic portrait of MCs but did not capture their dorsoventral heterogeneity, as it was restricted to dorsal MCs. To overcome this limitation, we employed TRAP after targeted microdissection of the dorsal and ventral hippocampus, enabling robust profiling of the subpopulation-specific translatome within the *Drd2*-expressing, MC-enriched sample. Our comparative molecular profiling revealed significant molecular heterogeneity along the dorsoventral axis, providing molecular insight into their distinct neurobiological properties and suggesting potential contributions to functional specialization within the hippocampus.

### Intrinsic excitability and synaptic responsiveness

Our findings reveal significant molecular heterogeneity in ion channel and neurotransmitter receptor genes along the dorsoventral axis, which may relate to differences in intrinsic excitability and synaptic responsiveness. The dorsal dataset exhibits an enrichment of various potassium (Kv, Kir, SK/BK, and K2P families) and voltage-gated calcium channel (P/Q-, R-, and L-type) genes (Extended Data Figs. 4-1, 4-3). These enriched channels likely facilitate high-frequency responsiveness and enhanced synaptic integration by providing a biophysical basis for rapid repolarization (Rudy and McBain, 2001; Gu et al., 2007) and dendritic signal amplification (Magee and Johnston, 1995). Consistent with this molecular profile, dorsal MCs display a higher frequency of spontaneous excitatory postsynaptic potentials (EPSPs) and larger maximal EPSPs amplitudes than ventral MCs (Jinno et al., 2003). Furthermore, the enriched expression of glutamate and GABA receptor subunit genes in dorsal population is consistent with strong dependence of dorsal MCs on synaptic drive, as their activity is abolished when glutamatergic and GABAergic inputs are pharmacologically blocked (Jinno et al., 2003). In contrast, ventral MCs exhibit intrinsic rhythmic bursting that persists even under complete synaptic blockade (Jinno et al., 2003). Recent studies have also reported that ventral MCs are enriched in neuropeptide signaling (Oh et al., 2026). Because the synthesis, transport, and release of neuropeptides during rhythmic bursting are energetically demanding processes, this physiological profile is consistent with our finding that ventral population shows enriched expression of genes involved in energy metabolism and vesicle exocytosis (Fig. 3*H*). Supporting this, our data show increased expression of *Stx1a*, which encodes a SNARE protein that mediates vesicle fusion (Fig. 3*I*). Given that *Stx1a*-deficient neurons retain fast synaptic transmission but exhibit impaired neuropeptide release (Fujiwara et al., 2006; Mishima et al., 2012), these findings suggest that ventral MCs may be particularly suited for neuromodulatory signaling, in contrast to the more input-driven fast synaptic signaling in dorsal MCs.

### Axon guidance and synaptic connectivity

We and others have demonstrated that MC subpopulations exhibit distinct axonal projection patterns along the dorsoventral axis (Botterill et al., 2021; Houser et al., 2021; Jeong et al., 2024b). Ventral MC axons primarily target the inner molecular layer (IML) of the DG throughout the hippocampus, while dorsal MC axons also target the dorsal IML but progressively shift innervation toward the middle molecular layer ventrally. This topographic projection is governed by complex molecular interactions involving axon guidance cues and cell adhesion molecules (Förster et al., 2006). Our translatomic data reveal differential expressions of key guidance molecules that may contribute to these subregion-specific connectivity patterns. The dorsal population expresses higher levels of Eph receptor subtypes (*Epha4*, *Epha5*, *Epha7*, *Ephb1*) and other guidance molecules (*Sema5a*, *PlexnA4*, and *Robo3*), while ventral population shows higher expression of Eph receptor ligands (*Efna1* and *Efnb2*; Fig. 3*G*). These molecular signatures align with repulsive axon guidance models. For instance, Epha5-expressing entorhinal axons are repelled from the Eph ligand-rich area; the IML of the DG (Stein et al., 1999) and Ephb1-expressing axons of retinal ganglion cells avoid Efnb2-expressing region, the midline of the optic chiasm (Williams et al., 2003). These differential patterns may reflect interactions between the differentially expressed guidance molecules within these subpopulations and the expression of their cognate ligands/receptors in target layers. As our data represent the adult state and axon guidance is primarily developmental, future studies on the expression dynamics of these molecules during hippocampal development are needed to fully elucidate their roles in establishing dorsoventral heterogeneity in MC connectivity.

Recent studies have established distinct dorsal and ventral MC roles in DG activity modulation (Jinde et al., 2012; Bui et al., 2018; Fredes et al., 2021; Li et al., 2022; Jeong et al., 2024a). Chemogenetic activation of dorsal MCs decreases, whereas activation of ventral MCs increases, c-Fos expression in the dorsal DG (Bauer et al., 2021; Fredes et al., 2021), suggesting that these subpopulations exert opposing net effects on DG activity. This functional differentiation is further supported by electrophysiological evidence showing that dorsal MCs exert a low excitation–inhibition (E/I) balance on local dorsal GCs but provide stronger excitatory input to distal ventral GCs, whereas ventral MCs reduce the E/I balance of local ventral GCs while conveying excitatory drive to distant dorsal GCs (Abdulmajeed et al., 2022), suggesting differential E/I control along the dorsoventral axis. Specificity and strength are shaped by molecular interactions mediated by cell adhesion molecules and synaptic assembly proteins expressed in both pre- and postsynaptic neurons (Südhof, 2018). Our molecular profiling provides a potential mechanistic basis for these differences, as the dorsal population is enriched for synaptogenic regulators such as Epha7 and Nrg3 (Fig. 3*G*), which are known to promote excitatory synapse formation onto hippocampal parvalbumin-positive basket cells (PV + BC; Beuter et al., 2016; Müller et al., 2018), which aligns with ultrastructural evidence that dorsal MC axons form a higher density of synapses onto PV + BC dendrites compared with ventral MC axons (Fredes et al., 2021). Altogether, these observations suggest that differential enrichment of various guidance and synaptic regulator molecules in dorsal and ventral MCs may provide a molecular substrate for divergent synaptic targeting. This, in turn, potentially leads to differential regulation of GC excitability via a differential E/I balance along the dorsoventral axis of the DG. Future studies targeting these subpopulation-specific molecules combined with layer-specific circuit analysis and quantitative electron microscopy will be essential to establish a causal link between the molecular distinctions and the relative dominance of postsynaptic targets for each MC subpopulation.

### Functional differentiation in hippocampal processing

The hippocampus exhibits clear functional heterogeneity along its dorsoventral axis, contributing differentially to cognitive versus emotional processing (Fanselow and Dong, 2010). MCs, with unique long-range associational projections spanning this axis, can integrate region-specific inputs and propagate them across these functionally distinct hippocampal domains (Scharfman, 2016), thereby facilitating functional associations across the dorsoventral axis of the DG. Our findings reveal dorsoventral molecular distinctions of *Drd2*-expressing MC–enriched populations, suggesting that their functional specialization may be associated with region-specific molecular signatures. Specifically, the dorsal population is enriched in genes related to synaptic transmission, excitatory signaling, and neuronal excitability, which might reflect the high-precision temporal encoding required for cognitive tasks. This is consistent with observation that dorsal MCs exhibit neural remapping in different environments and local cue, supporting their contribution to pattern separation process (Myers and Scharfman, 2009; GoodSmith et al., 2017; Nakazawa, 2017; Senzai and Buzsáki, 2017; GoodSmith et al., 2019; Jung et al., 2019; Galloni et al., 2022; Huang et al., 2024). Furthermore, dorsal MCs are crucial for rapid contextual discrimination (Jeong et al., 2020). Conversely, the ventral population preferentially expresses genes involved in metabolic pathways and mitochondrial function, which may support sustained neural activity observed under emotionally salient and stress-related conditions. This molecular profile aligns with reports of heightened ventral MC activity during anxiogenic exploration and their role in novelty detection to facilitate memory formation (Fredes et al., 2021; Wang et al., 2021). While further functional studies are needed to establish causal links, these distinct molecular signatures provide a plausible basis for the specialized contributions of these MC subpopulations to context-dependent information flow along the hippocampal axis.

### Disease vulnerability of MC subpopulations

MCs are vulnerable in many neurological and psychiatric disorders, including epilepsy, ischemia, traumatic brain injury, stress-related conditions, and Alzheimer's disease (AD; Crain et al., 1988; Buckmaster and Jongen-Rêlo, 1999; Santhakumar et al., 2000; Jiao and Nadler, 2007; Volz et al., 2011; Sloviter et al., 2012; Oh et al., 2020; Li et al., 2022; Jeong et al., 2024a). Our findings of distinct molecular signatures provide a molecular speculation on differential vulnerability. For instance, the dorsal population is enriched in genes associated with synaptic transmission and calcium signaling (Fig. 4*A*), which could potentially render them more susceptible to excitotoxicity (e.g., epilepsy, ischemia, and stress). This notion is consistent with studies showing substantial dorsal MC degeneration in temporal lobe epilepsy models, with relative preservation of ventral MCs (Huang and Houser, 2024). Conversely, the ventral population possesses molecular features that might contribute to relative resilience against calcium overloads. Their high expression of the calcium-buffering protein calretinin (*Calb2*) suggests a potential capacity for managing intracellular calcium loads (Schwaller, 2014). Furthermore, their enrichment in genes supporting oxidative phosphorylation suggests robust mitochondrial function, which is critical not only for energy production but also for calcium buffering and managing oxidative stress (Walters and Usachev, 2023). These molecular profiles align with maintained ventral MC activity under conditions of inescapable stress, while dorsal MC activity is suppressed (Jeong et al., 2024a). On the other hand, the enrichment of genes associated with mitochondrial function and energy metabolism in the ventral population (Fig. 4*C*) may underlie their specific susceptibility to metabolic disruption in neurodegenerative disorders including AD (Wilson et al., 2023). Supporting this idea, a recent study reported prominent accumulation of AD-like hyperphosphorylated and truncated tau proteins in ventral MCs, which impaired spatial memory (Li et al., 2022). Furthermore, our data show that *Cdk5* (Extended Data Fig. 3-1), a direct tau kinase whose hyperactivation drives abnormal tau hyperphosphorylation (Liu et al., 2016), is enriched in the ventral dataset, which may provide a potential link to the tau pathology. Future studies must elucidate how the molecular characteristics of MC subpopulations causally contribute to their differential vulnerability and disease responses.

### Limitations of the study

While providing significant insights, our study has several important limitations. First, we acknowledge the inherent limitations associated with the *Drd2*-Cre line, as we cannot entirely rule out minor translational signature contamination from off-target. Although this approach robustly labels MCs within the DG hilus, it can also capture a substantial subset of other *Drd2*-expressing hippocampal GABAergic interneurons. Additionally, despite targeted microdissection of the ventral subiculum, the potential for minor inclusion of the adjacent subicular tissue remains. Therefore, while the dorsoventral heterogeneity is strongly attributed to the MCs, which is supported by our histological evidence, the overall translatome data should be interpreted as representing a *Drd2*-expressing, MC-enriched population, with appropriate scientific caution. Second, the TRAP methodology itself imposes limitation, as it provides a focused view of the translatome (actively translating mRNA) but excludes nontranslating nuclear RNAs and may miss transcripts located in distal processes or those not actively bound to ribosomes. Unlike single-cell or single-nucleus RNA sequencing, which profile total or nuclear transcripts, TRAP captures only the ribosome-bound fraction. Complementary higher-purity approaches, such as single-cell RNA sequencing with rigorous cell-type classification, are warranted to capture the full extent of the regional and intraregional diversity within MCs. Third, our binary “dorsal/ventral” classification, based on anatomical dissection, may oversimplify the continuous septotemporal axis, and we acknowledge that further intraregional heterogeneity may exist within these subregions. Despite these limitations, our study provides a comprehensive basis for understanding the molecular heterogeneity of MC-enriched subpopulations along the dorsoventral axis, offering a foundation for future investigation.

## Synthesis

Reviewing Editor: Mark Sheffield, University of Chicago Division of the Biological Sciences Decisions are customarily a result of the Reviewing Editor and the peer reviewers coming together and discussing their recommendations until a consensus is reached. When revisions are invited, a fact-based synthesis statement explaining their decision and outlining what is needed to prepare a revision will be listed below. The following reviewer(s) agreed to reveal their identity: Cheng-Chang Lien. Note: If this manuscript was transferred from JNeurosci and a decision was made to accept the manuscript without peer review, a synthesis may not be available.

The reviewers and I agree that the study provides an important transcriptomic comparison of dorsal and ventral dentate gyrus cells and offers potentially valuable insights into mossy cell heterogeneity. However, serious concerns were raised about the specificity of the Drd2-Cre line for mossy cells, with known contamination by interneurons and subicular cells, especially when crossed with a reporter line. To address this without requiring extensive new experiments, we suggest you revise your terminology to refer to the population as "Drd2-expressing hippocampal cells" and explicitly acknowledge this limitation. Additional clarification of anatomical dissection, statistical approaches, and transcript validation methods is also needed before the manuscript can be considered further. All comments from the reviewers must be addressed and can be found below.

Reviewer 1:

This study by Jeong et al. aimed to create a transcriptional atlas of hilar mossy cells (MCs) along the dorsoventral axis of the dentate gyrus (DG) in the hippocampus and to propose possible molecular mechanisms underlying their functional specialization. Using translating ribosome affinity purification (TRAP) in Drd2-Cre;EGFP-L10a transgenic mice, the authors isolated Drd2 cell-specific mRNAs from the dorsal and ventral hippocampal regions for RNA sequencing.

It is important to note - and regrettable - that the Drd2-Cre line is not specific to MCs in the hilus, as Drd2-positive cells in the DG include both mossy cells and hilar interneurons. Furthermore, the authors' dissections included the subiculum, where Drd2 expression is abundant. Therefore, I refer to these cells as "Drd2 cells" rather than "MCs," as the authors describe. This is a critical issue that compromises the fidelity of their results and conclusions. The title of the manuscript should be rephrased to something like "Dorsoventral transcriptional atlas of hippocampal Drd2 cells reveals molecular heterogeneity underlying functional specialization."

The study identified 1,442 genes enriched in dorsal Drd2 cells and 1,337 genes enriched in ventral Drd2 cells, revealing profound transcriptional heterogeneity. Pathway analyses showed that dorsal Drd2 cells preferentially express genes associated with neuronal connectivity, synaptic transmission, ion channels, and neurotransmitter receptors, suggesting roles in cognitive processing. In contrast, ventral Drd2 cells were enriched for genes involved in metabolic regulation, oxidative phosphorylation, and cellular homeostasis, indicating specialization for sustained activity and affective processing. Furthermore, analyses predicted distinct upstream regulatory networks, with dorsal Drd2 cells regulated by factors such as Tcf7L2 and Egr2, and ventral Drd2 cells by Ppargc1a and Nfe2l2. It is disappointing, however, that these findings cannot specifically account for the distinct functions of MCs in the dorsal and ventral poles.

Overall, while this work attempts to provide a molecular framework for understanding the function of Drd2 cells (note: I disagree with referring to them as "MCs") within hippocampal circuits and offers a foundation for future studies, several major concerns remain:

Major Concerns

1. The Drd2-Cre line used by the authors is not specific for MCs, as Drd2-positive cells in the hilus include both mossy cells and interneurons, and Drd2-Cre-expressing cells are also abundant in the subiculum. Recent work by Godino et al. (2025) showed that GABAergic neurons also express Drd2 receptors. For MC specificity, combination of viral strategy and the Crlr-Cre line is currently most specific approach to MCs if precise AAV injection in the DG (Wang et al., 2021; Abdulmajeed et al., 2022). Thus, I remain unconvinced by the authors' claim of MC-selective targeting and their interpretations.

2. Although the dorsal hippocampus is involved in spatial cognition and the ventral hippocampus in affective behaviors, the current data do not directly address MCs' functional roles in these domains. Some gene expression patterns are inconsistent with known data and lack protein or pharmacological validation. For example, Figure 4B shows DRD5 and GRM5 enrichment in dorsal MCs, findings unconfirmed at the cellular level. Given mGluR5's critical role in DG functions, its preferential expression in dorsal MCs should be validated by in situ hybridization (ISH) and immunohistochemistry (IHC).

3. The manuscript lacks clear definitions of dorsal and ventral DG regions. The authors should dissect the DG into dorsal, intermediate, and ventral parts, using only dorsal and ventral segments for TRAP analysis to avoid confounding. Dissections should be performed under a stereomicroscope to prevent contamination of the subiculum.

4. The identified genes do not sufficiently interpret or explain the distinct functional roles of dorsal and ventral MCs, limiting the translational impact of the findings.

5. All ISH data presented are adopted from the Allen Brain Atlas, which lacks cell-type specificity. Confirmation based solely on this database is inadequate. The authors should perform co-immunostaining to verify whether the protein expression of these genes in MCs (GluR2/3-positive cells) shows dorsoventral differences. At minimum, IHC validation of Calb2 and Cyp26b1 should be included.

6. The study should include markers not expected to be enriched in dorsal or ventral MCs, such as GAD65 or GAD67, as negative controls. Additionally, qPCR validation should be performed, and other negative markers considered.

Minor Concerns:

1. For all ISH data adopted from the Allen Brain Atlas, the authors should provide the experiment number, probe name, section number, and scale bar in the figure legends to ensure data transparency.

2. In Figures 1B and 1C, the nuclear stain is labeled as DRAQ5, but the Methods indicate the use of DAPI. Please clarify which nuclear stain was actually used.

3. A brief discussion of genes shared across dorsal and ventral MCs, and distinct from other hippocampal neurons, could help highlight core mossy cell features. Such genes may be identified using existing transcriptomic resources (e.g., Cembrowski et al., 2016; Allen Brain Atlas).

Reviewer 2:

This manuscript is about the transcripts in dorsal vs. ventral dentate gyrus mossy cells. The paper is important because mossy cells play an important role in the dentate gyrus, and it is clear there are differences between dorsal and ventral mossy cells.

The authors identify many differences between dorsal and ventral mossy cells that are very interesting. Overall the paper is written well and the data are presented well. There are a few concerns that are important and many minor concerns.

Important:

Are these nuclear or cytoplasmic transcripts, and what about transcripts in dendrites and axons? I think the transcripts in dendrites and axons were not studied, and if that is true a limitation should be added. It is minor because most studies of this kind do not consider dendrites and axons. It is more important to mention whether the transcripts are only those from the nucleus (sn-RNA seq) or cytoplasmic and nuclear (sc-RNA seq). Along these lines, please clarify how TRAP differs from other types of studies that use sc-RNA seq or sn-RNA seq. For example, explain the mouse line with more information. Throughout the paper, explain abbreviations.

The authors note that there is a limitation about how precise they discriminated between dorsal and ventral, which is good. But there is no information at all how the cut was made to divide the two portions. This is very important because the schematic at the end shows the dorsal portion is somewhat smaller than the ventral portion.

The paper relies on markers of mossy cells but it is not clear what proportion of mossy cells express each marker. For example, not all mossy cells in ventral dentate gyrus express calretinin and it varies from mouse to mouse. Some numbers are needed to prove each marker identified almost all mossy cells.

It is important to note how the granule cell layer and hilus were divided because calretinin stains young granule cells at the border, potentially contaminating the calretinin-stained population of mossy cells.

Lines 249-251 are examples of subjective assessments. Please increase the rigor here and elsewhere.

Please increase the information about statistics. For example, if all stats were two way anova then one has to know the data meet the characteristics of parametric tests.

Line 75

Please also consider references to work of many others:

PMID: 38909362

PMID: 35221956

PMID: 31641051

PMID: 31267019

PMID: 28279358

PMID: 28132828

PMID: 28132824

PMID: 18958849

Line 82

The reference to Ratzliff et al 2004 seems out of place here -please check it is appropriate

Please validate the anitbodies

Why was 5 mice enough?

Areas of the text that need clarification:

30 min to minimize blood trace...what is "blood trace"?

Superasin...please explain

motor-driven Teflon glass homogenizer...what is that item and vendor?

to pellet insolubilized materials...do the authors mean to separate insoluble material as a pellet?

50 μg each of the clones 19C8 and 19F7. This is useful but one needs to know antibody concentration.

protein L...please explain

using an end-over-end rotator...please explain what 'end-over-end' means

for 16 hours Page 13/27 at 4 {degree sign}C... it seems "page13/27" is from another paper

with in-column DNase digestion...Please explain

Line 157 Quality Number (RQN) greater than eight (out of ten) were used for qPCR and RNA-seq. How was this decision made? It seems arbitrary

single melting curve patterns...Please explain

GO and KEGG... Explain all abbreviations after their first use, then use the abbreviation afterwards

I realize most papers of this kind do not confirm accuracy of the analytical tools but it would be great if the authors could do that. For example, for the software tools used, was there any check they were accurate?

Pathways with z-score> 2 were considered to be significantly activated in either dorsal or ventral MCs. How was this decision made? It seems arbitrary

This statement seems like an oversimplification.

"Dorsal MCs exert net inhibition on GCs via GABAergic interneurons, while ventral MCs provide net excitation through direct GC synapses (Fredes et al., 2021)." The paper that is cited is excellent, but it is not as clear as stated. One only knows that effects of manipulating dorsal MCs changed c-Fos.

Please also discuss work from Lien's paper.

PMID: 35671148

I think reference Grain et al 1988 should be Crain et al. 1988. Note they did not distinguish mossy cells from other hilar neurons.

PMID: 2464145

Also add references to other work showing mossy cells are vulnerable in epilepsy models from Sloviter:

PMID: 22787603

39637110

Nadler:

PMID: 17475251

Buckmaster:

PMID: 10531454
