# Supplementary figures and images for: Translational Profiling of Drd2-Expressing Populations Reveals Molecular Heterogeneity of Dentate Gyrus Mossy Cells along the Dorsoventral Axis
Source: eNeuro. 2026 Jul 14;13(7):ENEURO.0236-25.2026. doi: 10.1523/ENEURO.0236-25.2026 (PMC13379367; doi:10.1523/ENEURO.0236-25.2026)

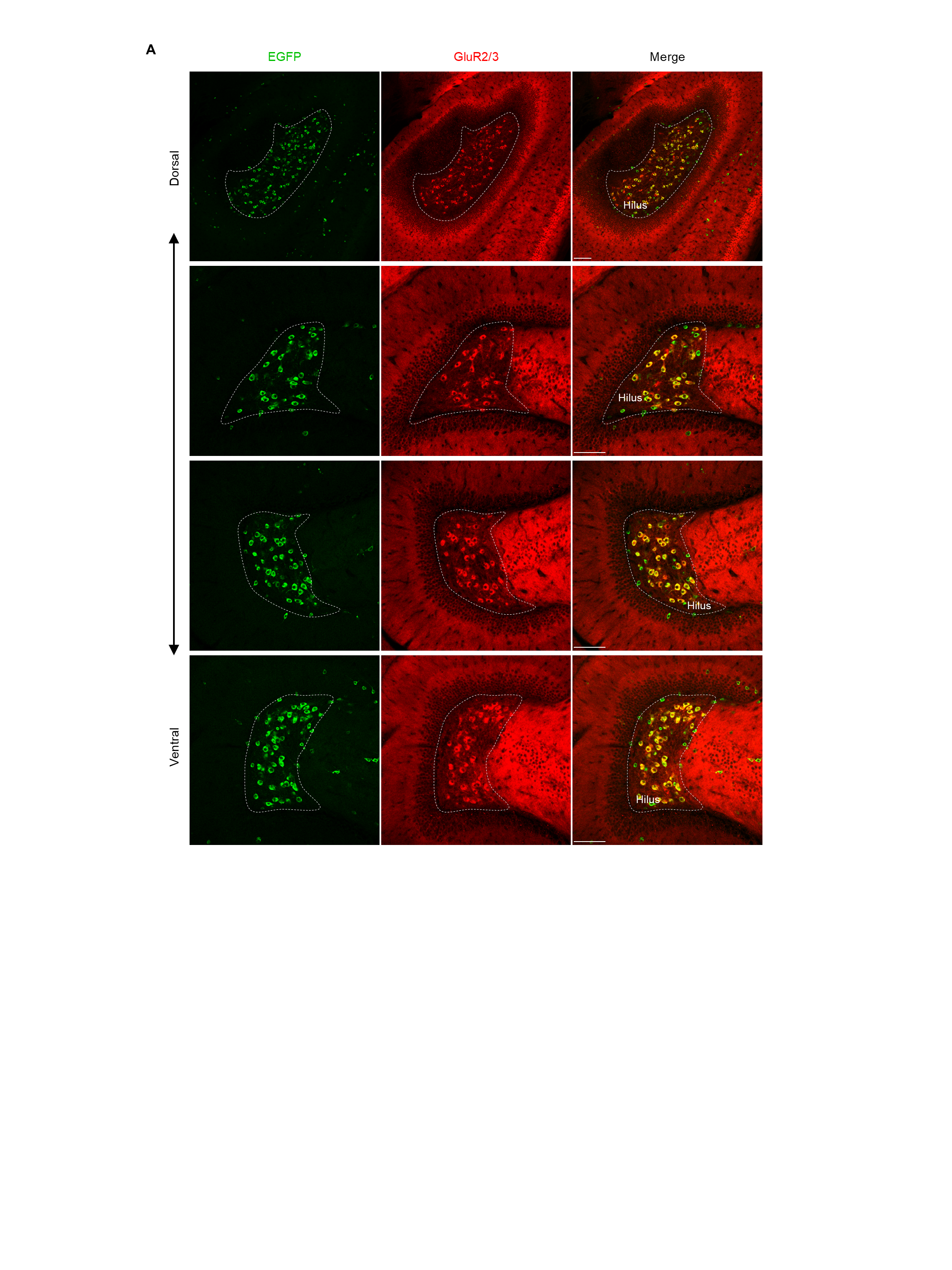

Supplement: Figure 1-1 — Selective expression of EGFP-L10a in MCs across the dorsoventral axis of the DG in MC-enriched TRAP mice. (A) Representative horizontal sections from Drd2-TRAP mice show selective expression of the EGFP-L10 in MCs along the dorsoventral axis of the DG. Immunohistochemistry for GluR2/3 (red), a marker for MCs, confirms colocalization with EGFP-L10a in the hilar region. White dashed lines outline the hilus. Scale bars, 100 μm. Download Figure 1-1, TIF file. [file eneuro-13-ENEURO.0236-25.2026-s001.tif]

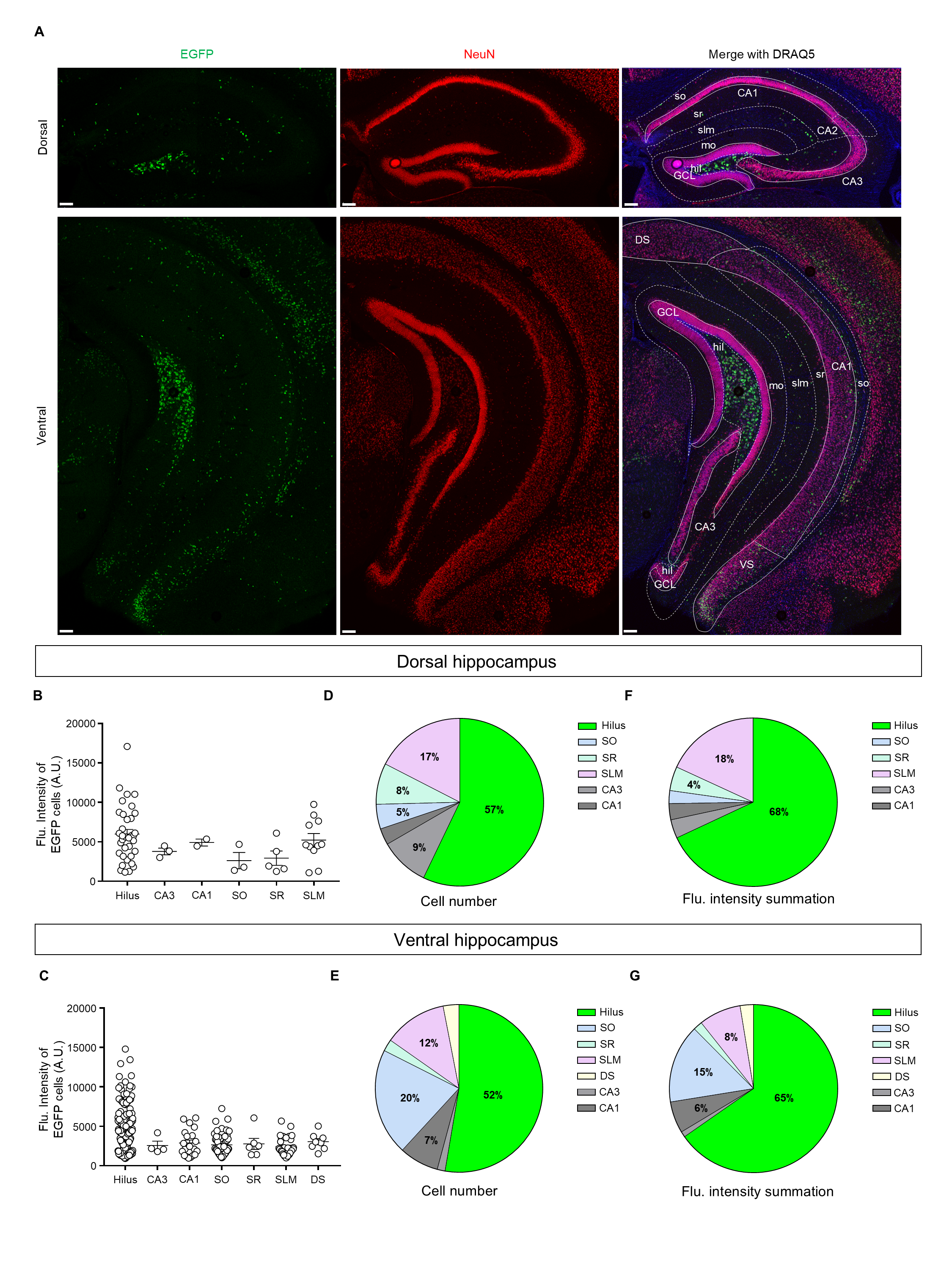

Supplement: Figure 1-2 — EGFP-L10a expressions along the dorsoventral axis of the hippocampus of MC-enriched TRAP mice. (A) Representative fluorescence images showing the distribution of EGFP-L10a (green) and NeuN (red) in the dorsal (top row) and ventral (bottom row) hippocampus. In both regions, strong EGFP-L10a expression is observed in MCs in the hilus. Sparse expression is also detected in interneurons located in CA1 so, sr, and slm. (B and C) Single-cell EGFP-L10a fluorescent intensity in the dorsal (B) and ventral (C) hippocampal subregions. Fluorescent intensity was calculated as pixel area multiplied by intensity per cell. (D and E) Pie chart showing the proportional distribution of EGFP-L10a positive cell number across the dorsal (D) and ventral (E) hippocampal subregions. For the dorsal hippocampus. (F and G) Pie chart showing total EGFP-L10a fluorescent intensity in each hippocampal subregion of the dorsal (F) and ventral (G) hippocampus. CA1–3, Cornu Ammonis areas; DG, dentate gyrus; GCL, granule cell layer; hil, hilus; mo, molecular layer; slm, stratum lacunosum-moleculare; sr, stratum radiatum; so, stratum oriens; DS, dorsal subiculum; VS, ventral subiculum. Scale bars, 100 µm. Download Figure 1-2, TIF file. [file eneuro-13-ENEURO.0236-25.2026-s004.tif]

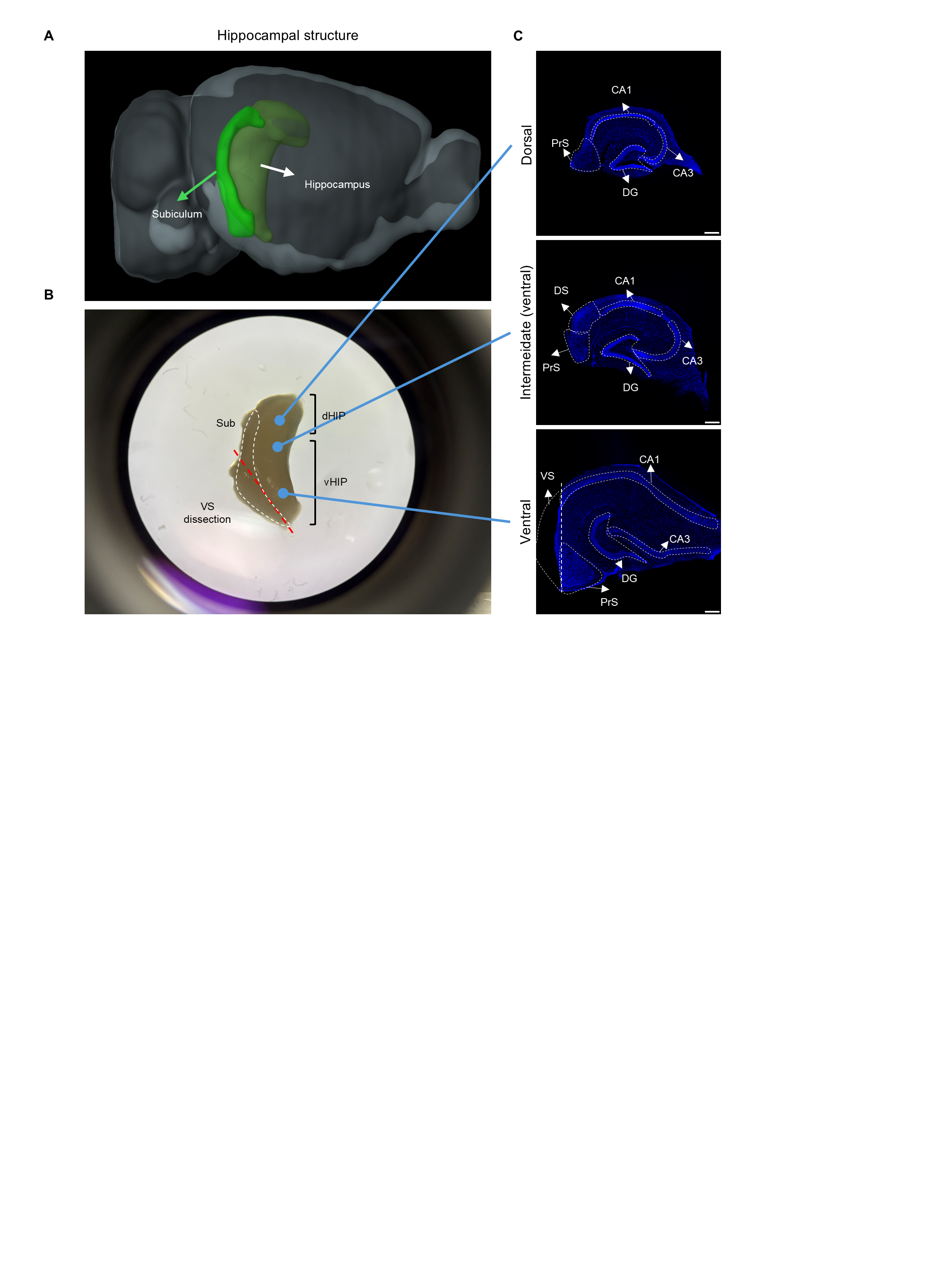

Supplement: Figure 1-3 — Anatomical validation of ventral subiculum microdissection. (A) Schematic illustration of hippocampal anatomy showing the location of the subiculum relative to adjacent hippocampal subregions. (B) Representative image of microdissection of the ventral subiculum from the hippocampus performed under a dissecting microscope. The ventral subiculum was anatomically identified and carefully isolated to minimize contamination from adjacent hippocampal regions. (C) Representative longitudinal hippocampal sections along the dorsoventral axis. dHIP, dorsal hippocampus; vHIP, ventral hippocampus; CA1–3, Cornu Ammonis areas; DG, dentate gyrus; Sub, Subiculum; DS, dorsal subiculum; VS, ventral subiculum; PrS, presubiculum. Scale bars, 200 µm. Download Figure 1-3, TIF file. [file eneuro-13-ENEURO.0236-25.2026-s005.tif]

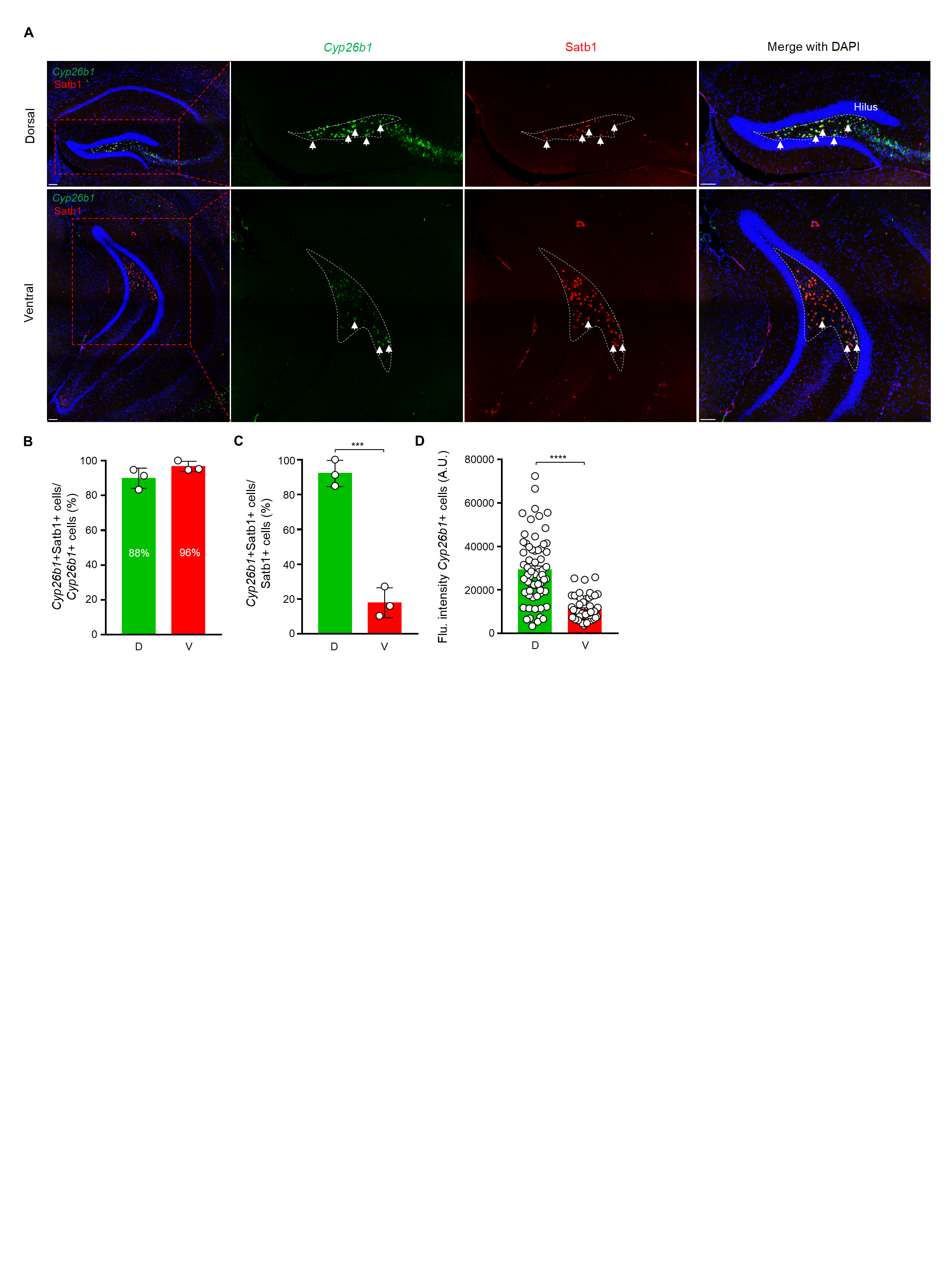

Supplement: Figure 1-4 — Histological validation of Cyp26b1 gene as a dorsal MC marker. (A) Representative images showing selective expression of Cyp26b1 gene in MCs along the dorsoventral axis of the DG. In situ hybridization (ISH) for Cyp26b1 mRNA (green) was combined with immunohistochemistry for the MC marker Satb1 protein (red) in the dorsal and ventral hippocampus. Arrowheads indicate Cyp26b1 ISH signals colocalized with Satb1 immunoreactivity in the hilus (outlined by white dashed lines). (B and C) Quantification of MC specificity (B) and coverage (C) of Cyp26b1expressing neurons colocalized with Satb1 along the dorsoventral axis of the DG (n = 3 mice). (Unpaired two-tailed Student’s t-test, ***p < 0.001). (C) Quantification of individual Cyp26b1 fluorescent intensity in the dorsal and ventral DG (Unpaired two-tailed Student’s t-test, ***p < 0.0001). Cells were identified and quantified using threshold-based detection of fluorescent signals. Fluorescent intensity was calculated as the product of the pixel area and mean intensity per cell. Scale bars, 100 µm. Download Figure 1-4, TIF file. [file eneuro-13-ENEURO.0236-25.2026-s008.tif]

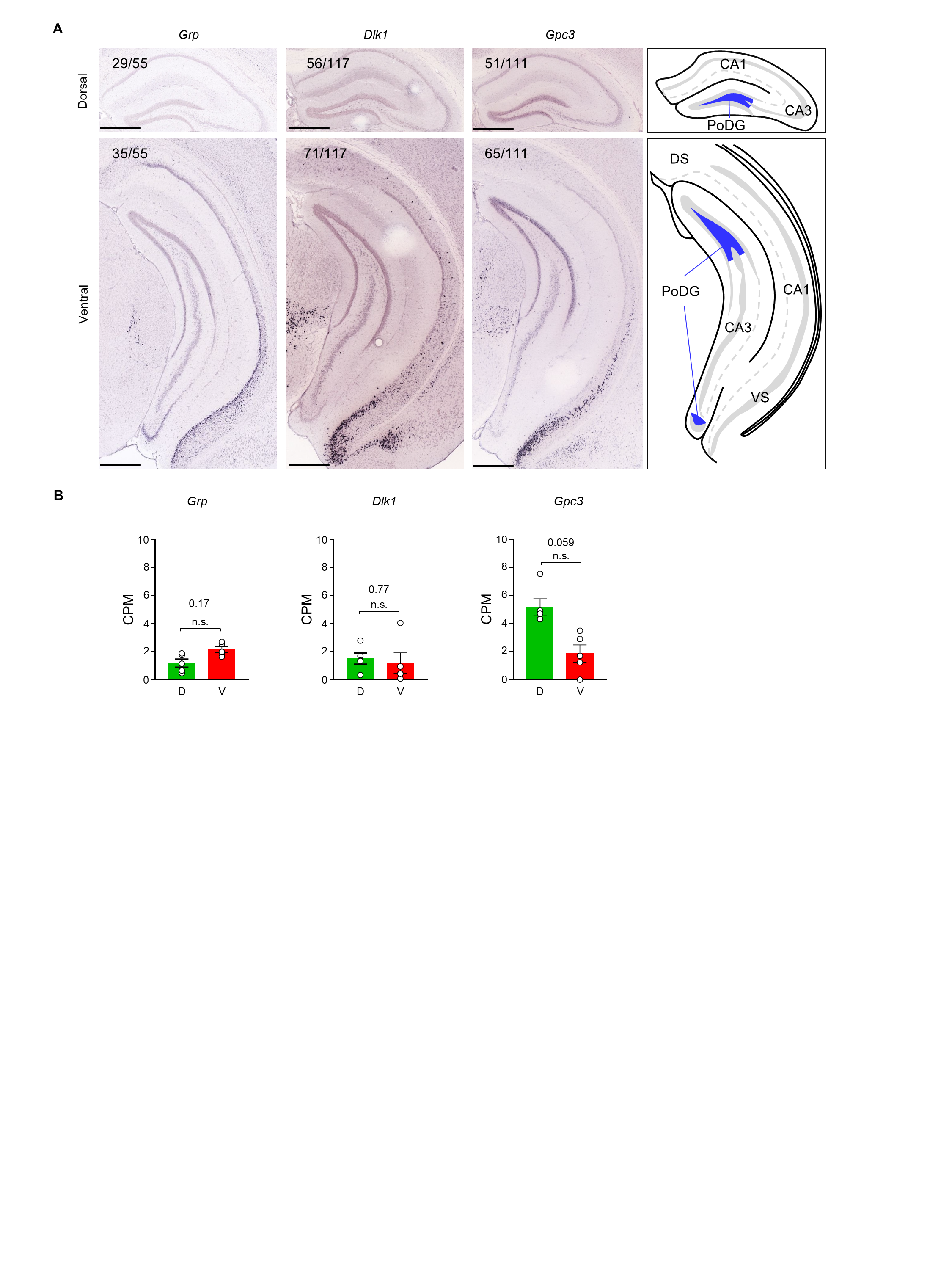

Supplement: Figure 2-1 — Quantitative TRAP-seq validation of ventral subiculum marker genes. (A) Representative in situ hybridization images showing expression patterns of ventral subiculum marker genes (Grp, Dlk1, Gpc3) in the dorsal and ventral hippocampus. (B) Quantification of marker gene expression in dorsal (green) and ventral (red) TRAP-seq datasets. Notably, transcript levels of these ventral subiculum markers are detected low in both dorsal or ventral TRAP-seq datasets and even Gpc3 seems de enriched in ventral dataset as compared to dorsal one (FDR-adjusted p > 0.05). Grp, experiment: 1363, probe: RP_Baylor_103371 – coronal, sections: 29/55 (dorsal) and 35/55 (ventral); Dlk1, experiment: 71587885, probe: RP_050725_03_A10 – coronal, sections: 56/117 (dorsal) and 71/117 (ventral); Gpc3, experiment: 71020431, probe: RP_050329_01_C10 – coronal, sections: 51/111 (dorsal) and 65/111 (ventral). PoDG, polymorphic layer of the dentate gyrus; DS, dorsal subiculum; VS, ventral subiculum; DG, dentate gyrus; CA1–3, Cornu Ammonis areas. Scale bars, 600 µm. Download Figure 2-1, TIF file. [file eneuro-13-ENEURO.0236-25.2026-s002.tif]

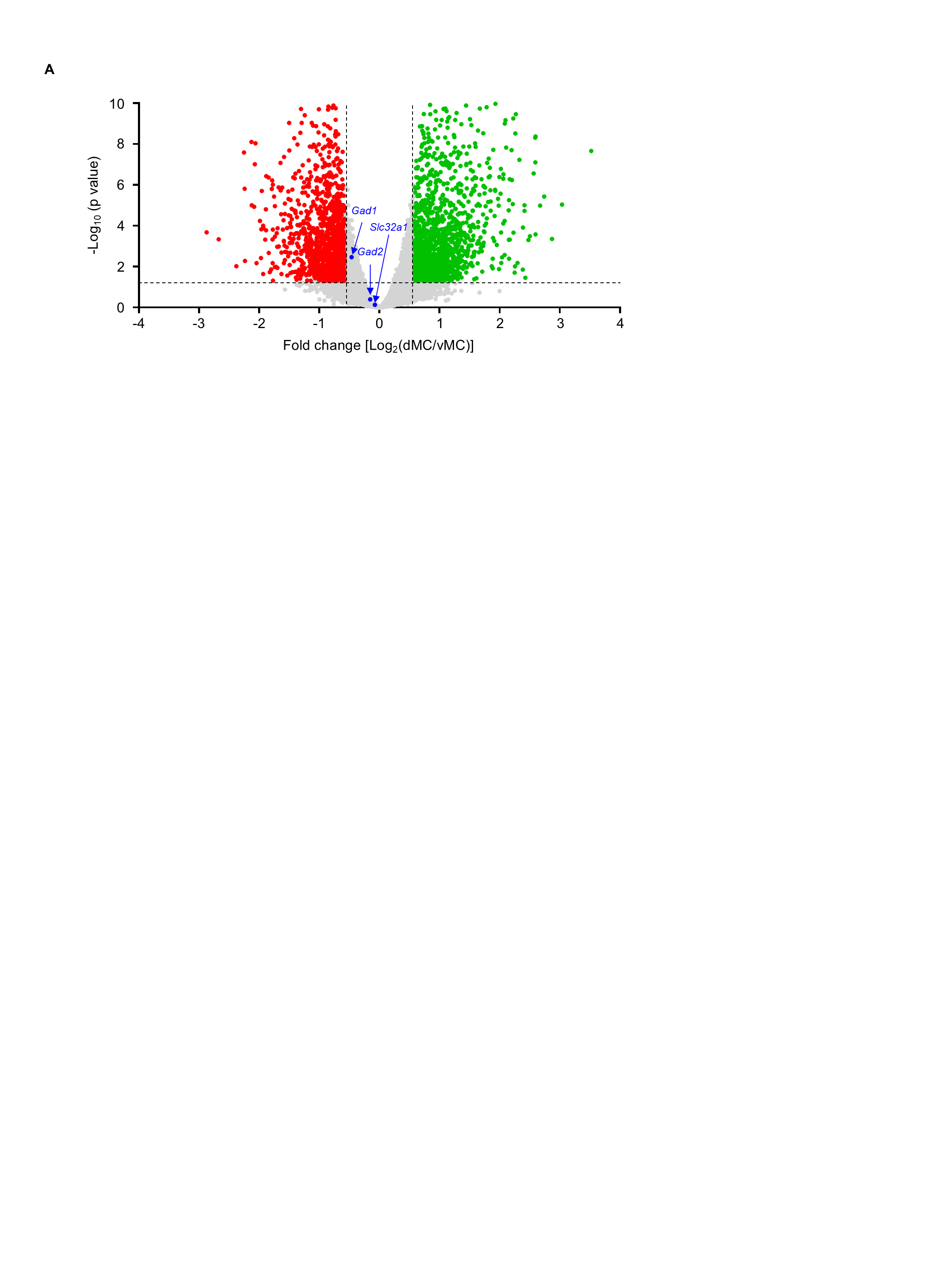

Supplement: Figure 2-2 — Comparable expressions of GABAergic interneuron markers between dorsal and ventral MC-enriched populations (A) Volcano plot showing that GABAergic interneuron marker genes are not significantly enrichment between dorsal and ventral MC-enriched samples. Red and green dots represent genes enriched in ventral and dorsal MC-enriched samples, respectively, while gray dots indicate non-differentially expressed genes. Blue labels highlight GABAergic interneuron markers (Gad1, Gad2, and Slc32a1), which showed no significant differences between dorsal and ventral MC-enriched samples. Download Figure 2-2, TIF file. [file eneuro-13-ENEURO.0236-25.2026-s006.tif]

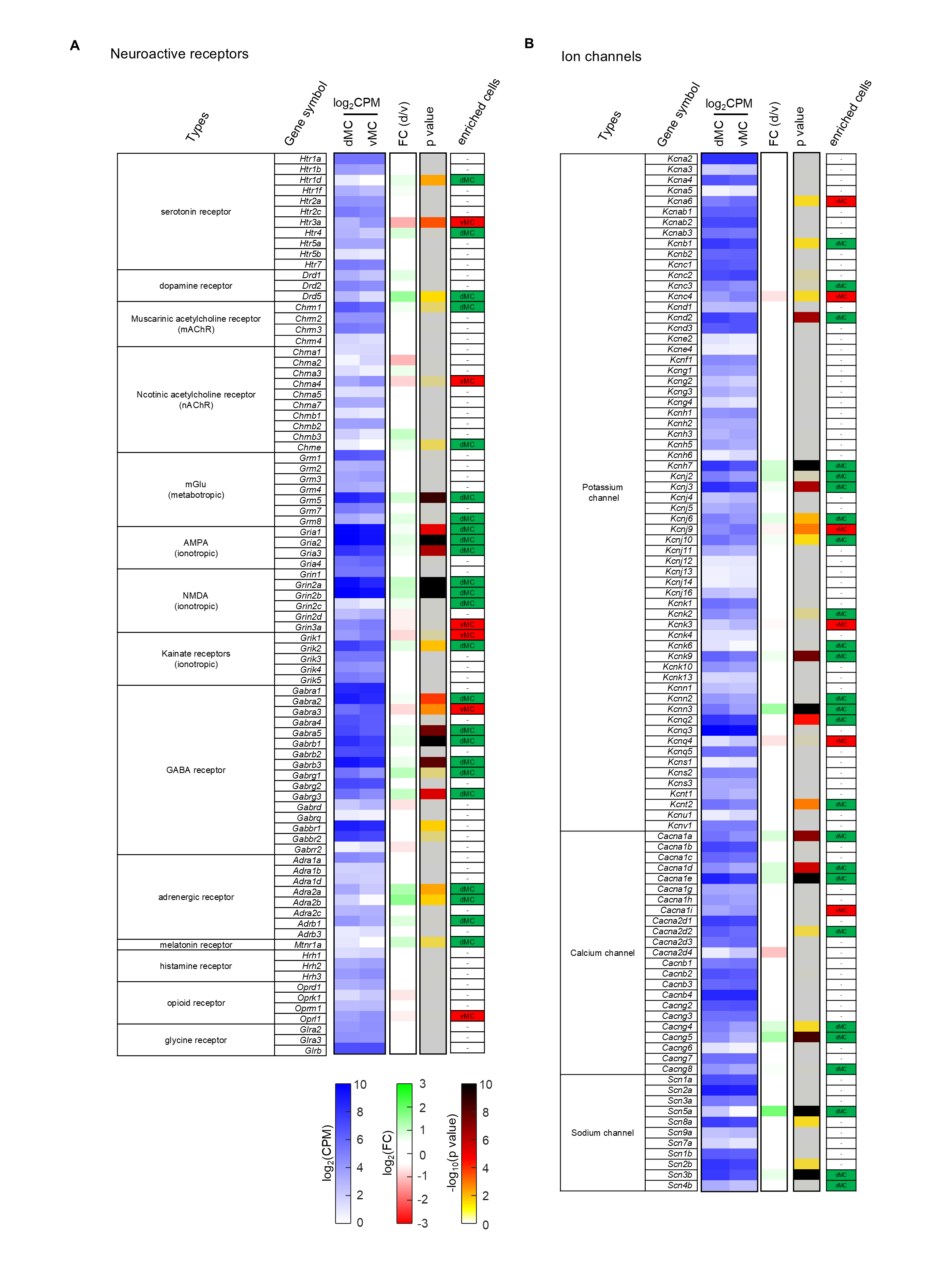

Supplement: Figure 4-1 — Distribution of Neurotransmitter receptors and Ion channels in MC-enriched population. (A and B) Heatmaps showing the expression pattern of DEGs in neuroactive receptors (A) and ion channels (B) in dorsal and ventral MCs. Functional categories for each gene are indicated. Raw expression levels are presented as log₂-transformed counts per million (log2[CPM]). Adjacent heatmaps display the corresponding log₂ fold changes (log2[FC]) and adjusted p-values (log10[adjusted p-value] for each gene, using distinct color gradients. Download Figure 4-1, TIF file. [file eneuro-13-ENEURO.0236-25.2026-s007.tif]
